# Machine Learning Methods for Predicting Human-Adaptive Influenza A Viruses Based on Viral Nucleotide Compositions

**DOI:** 10.1093/molbev/msz276

**Published:** 2019-11-21

**Authors:** Jing Li, Sen Zhang, Bo Li, Yi Hu, Xiao-Ping Kang, Xiao-Yan Wu, Meng-Ting Huang, Yu-Chang Li, Zhong-Peng Zhao, Cheng-Feng Qin, Tao Jiang

**Affiliations:** 1 Department of Virology, State Key Laboratory of Pathogen and Biosecurity, Beijing Institute of Microbiology and Epidemiology, Beijing, China; 2 Department of Clinical Laboratory, the Fifth Medical Centre of Chinese PLA General Hospital, Beijing, China; 3 Graduate School, Anhui Medical University, Hefei, China; 4 Department of Infection and Immunology, State Key Laboratory of Pathogen and Biosecurity, Beijing Institute of Microbiology and Epidemiology, Beijing, China

**Keywords:** human adaptation, influenza A viruses (IAVs), genomic nucleotide composition, machine learning (ML), dinucleotide

## Abstract

Each influenza pandemic was caused at least partly by avian- and/or swine-origin influenza A viruses (IAVs). The timing of and the potential IAVs involved in the next pandemic are currently unpredictable. We aim to build machine learning (ML) models to predict human-adaptive IAV nucleotide composition. A total of 217,549 IAV full-length coding sequences of the PB2 (polymerase basic protein-2), PB1, PA (polymerase acidic protein), HA (hemagglutinin), NP (nucleoprotein), and NA (neuraminidase) segments were decomposed for their codon position-based mononucleotides (12 nts) and dinucleotides (48 dnts). A total of 68,742 human sequences and 68,739 avian sequences (1:1) were resampled to characterize the human adaptation-associated (d)nts with principal component analysis (PCA) and other ML models. Then, the human adaptation of IAV sequences was predicted based on the characterized (d)nts. Respectively, 9, 12, 11, 13, 10 and 9 human-adaptive (d)nts were optimized for the six segments. PCA and hierarchical clustering analysis revealed the linear separability of the optimized (d)nts between the human-adaptive and avian-adaptive sets. The results of the confusion matrix and the area under the receiver operating characteristic curve indicated a high performance of the ML models to predict human adaptation of IAVs. Our model performed well in predicting the human adaptation of the swine/avian IAVs before and after the 2009 H1N1 pandemic. In conclusion, we identified the human adaptation-associated genomic composition of IAV segments. ML models for IAV human adaptation prediction using large IAV genomic data sets can facilitate the identification of key viral factors that affect virus transmission/pathogenicity. Most importantly, it allows the prediction of pandemic influenza.

## Introduction

Type A influenza viruses (IAVs) infect a wide range of avian and mammalian hosts, generally with species specificity. Avian influenza viruses (AIVs) typically exist in natural reservoirs, waterfowl, and shorebirds (Yoon *et al.* 2014), which mostly cause subclinical bird infection (Webster et al. 1978; Long et al. 2019). AIVs sporadically infect mammalian hosts, such as swine (Pensaert et al. 1981), human beings (Subbarao and Katz 2000; de Jong et al. 2006; Lam et al. 2013), and other mammals (White 2013; Lee et al. 2017) and are incapable of intraspecies transmission (Tran et al. 2004; Maines et al. 2006; Long et al. 2019). However, the high frequency of mutation and segment recombination endows AIVs with the chance to obtain human-adaptive genomes, which pose a high pandemic risk. Notably, swine adaptation and swine-adapted IAVs are closely related to human pandemics. All of the last five recorded influenza pandemics were caused by avian-origin, swine-origin, or reassortant IAVs (Reid et al. 2004; Kislinger et al. 2006; Bragstad et al. 2011; Long et al. 2019). Thus, it is of great importance to predict the adaptation of avian or swine IAVs to humans.

Human-adaptive IAVs are capable of infecting and causing disease in humans easily and of spreading among human populations efficiently. To date, H3N2 and H1N1 (including seasonal H1N1 and A(H1N1)pdm09) are dominant human-adaptive IAV subtypes that cause epidemics in humans (Ren et al. 2016). H5N1, H7N9, and other IAV subtypes occasionally infect humans but are not yet capable of spreading in human populations (Yang et al. 2007; Rudge and Coker 2013; Hu et al. 2014; Deng et al. 2017). Laboratory studies have identified numerous viral determinants that are associated with the human adaptation of IAVs via mediating receptor binding, regulating the virus’s replication cycle, and antagonizing host immunity (Taubenberger and Kash 2010; Bouvier 2015; Long et al. 2019). However, there are no universal human adaptation determinants for IAVs.

Gene sequencing technology and machine/deep learning methods have facilitated virus sequence identification of a considerably large data set, including IAVs. Machine learning (ML) methods have recently demonstrated their effectiveness in multiple disciplinary fields, including virology. The distinct host tropism protein signatures of IAVs (Eng et al. 2016), the zoonotic risk of various viruses (Eng et al. 2017), and even the avian-to-human transmission risk of IAVs (Qiang et al. 2018) have been recognized. The host dependence of mononucleotides (nts) and tetranucleotide compositions of influenza viruses has also been studied with ML methods (Iwasaki et al. 2013). Notably, the prominent role of dinucleotides in virus genomes has been implicated in both experimental and computational reports. Viral dinucleotides are targets for the host innate immune system (Takata et al. 2017), and they independently regulate the virulence (Atkinson et al. 2014; Tulloch et al. 2014) and replication (Witteveldt et al. 2016) of IAV viruses. Species-specific (Glass et al. 2007) and virus-family-specific (Di Giallonardo et al. 2017) dinucleotide compositions have also been computationally recognized. More recently, the dinucleotide composition in RNA virus genomes accurately predicts viral reservoir hosts and arthropod vectors using ML methods (Babayan et al. 2018). Therefore, we hypothesize that genomic dinucleotide composition is another crucial genomic feature for influenza viruses, which is most likely amino acid independent, and may be useful for characterizing the human adaptation feature of IAVs.

In the present study, 60 types of mono- and dinucleotide compositions were analyzed based on the nucleotide position within a codon in the full-length coding sequences of the first six genomic segments of IAVs: PB2 (polymerase basic protein 2), PB1, PA (polymerase acidic protein), HA (hemagglutinin), NP (nucleoprotein), and NA (neuraminidase). These (d)nts were optimized based on their relative importance, with principal component analysis (PCA) and support vector classifier (SVC) methods. Then, ML models of gradient-boosted regression trees (GBRT), multilayer perceptron (MLP) classifier, random forest (RF) classifier, and SVC were built to analyze and predict the human adaptation of human-, swine-, or avian-origin IAVs. Our models perform well in predicting human-adaptive swine or avian IAVs.

## Results

### Prediction Pipeline and Data Processing of the Genomic Nucleotide Composition in IAVs

**FIg. 1.**
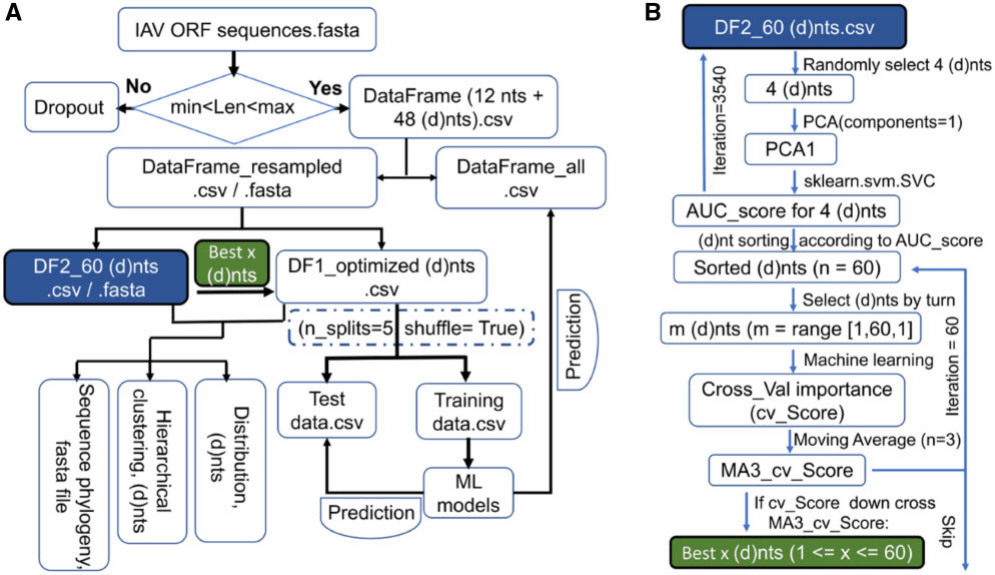
The workflow of data processing, feature optimization, and model construction. (*A*) The workflow of data processing and model construction. Original ORF sequences within the length range were utilized for codon dependently counting 12 mononucleotides (nts) and 48 dinucleotides (dnts). The counting file for the 60 (d)nts for the PB2, PB1, PA, HA, NP, or NA segments was randomly resampled to maintain a balanced distribution of human and avian sequences. The (d)nt composition distribution and the sequence phylogeny were analyzed, and then the randomly split (d)nt counting data were utilized for model building and testing. (*B*) Workflow of the feature extraction with PCA/ SVC methods. The 60 (d)nts were sorted according to the score (AUC) from the SVC analysis, with the PCA-extracted principal component from the randomly selected (d)nt counting information. Accumulated (d)nts in the sorted list were analyzed with ML models, with the cross of the AUC score curve down its MA score (*n* = 3) as the threshold of the best number of (d)nts. “DF2_60 (d)nts.csv” for the (d)nt sorting and “Best x (d)nts” for data optimization were, respectively, labeled with blue and green text boxes filled in both (*A*) and (*B*).

As the workflow diagram in figure 1*A* shows, data wrangling was performed for IAV open reading frame (ORF) sequences. Twelve types of mononucleotides (nts) and 48 types of dinucleotides (dnts) in the ORF were counted for all the sequence samples. The phylogeny of the sample ORF sequences, the hierarchical clustering of sequence samples based on the 60 (d)nts,, and the distribution of the sequence samples, in each type of sequence label, were analyzed (fig. 1*A*). The 60 (d)nts were sorted based on their importance (cross-validation score, cv_Score) for the classifier with PCA and SVC methods (fig. 1*B*), and the best (d)nts (fig. 1*B*), which were optimized with ML approaches from the sorted (d)nts, were utilized for the final data optimization and the final prediction (fig. 1*B*).

In total, 226,183 full-length coding sequences for the first six segments (PB2, PB1, PA, HA, NP, and NA), available up to December 31, 2018, had skewed distributions for the labels of country/area, host, subtype, segment, or year. In total, 8,634 sequences were dropped, beyond the length range had repeated sequence IDs (supplementary table 1, Supplementary Material online). The remaining 217,549 sequences were still predominantly from the United States for the country/area and from 2009 to 2018 for the year (supplementary fig. 1, Supplementary Material online). Random resampling was performed to maintain a ratio of approximately 1:1 for the sequences from the United States and mainland China, the second largest influenza sample country. The resampled 83,980 avian and human sequences available for feature extraction and model building were dominantly from North America and East and Southeast Asia, particularly from the United States and China (supplementary fig. 2*A*, Supplementary Material online); the sample distributions of the different types of hosts, subtypes, segments and years are indicated (supplementary fig. 2*B*–*F*, Supplementary Material online). The 34,990 swine IAV sequences were not included in the training data because of their double biological adaptation to both human and avian hosts.

### The Characterization of the Human Adaptation-Associated Nucleotide Composition of IAV Sequences

The (d)nt composition was counted and compared within and between species based on the profile of relative dinucleotide abundance values according to previous reports (Karlin and Mrazek 1997). Hierarchical clustering was performed for 3.59–5.01‰ (59–61) randomly sampled sequences from each segment sequence set according to the (d)nt composition. The majority of human and avian IAVs were not clustered into human and avian branches, respectively, for the PB1, PA, HA, and NA segments (supplementary fig. 3, Supplementary Material online), and these selected sequence samples were not clustered into human and avian groups in a phylogeny tree (supplementary fig. 4, Supplementary Material online). Additionally, a PCA transversion of the 60 (d)nts was performed to evaluate the linear separability between major human sequences and avian sequences. There was no such separability in the principal component 1 or principal component 2 of the 60 (d)nts in the PB2, PA, HA, or NA segments; only the PB1 and NP segments were separable for both groups of subtypes for principal component 1 (supplementary fig. 5, Supplementary Material online).

An ML analysis combining PCA and SVC was performed to characterize the human adaptation-associated nucleotide composition of IAVs from the 60 (d)nts. As the workflow (fig. 1*B*) shows, 3,540 iterations of PCA/SVC analysis (guaranteeing more than 200 repeat analyses for each (d)nt) (supplementary fig. 6, Supplementary Material online) were performed to reduce every four (d)nts into one principal component, which was then utilized for the SVC for avian and human IAV sequences. The statistical analysis results of the cross-validation score (Cross_val score) from the PCA/SVC for the 60 (d)nts of each segment are listed in supplementary tables 2–7, Supplementary Material online. The 60 (d)nts were sorted according to the mean Cross_val score, in other words, based on the feature importance (supplementary fig. 7*A*–*F*, Supplementary Material online, for the six segments). The plotting of the relative (d)nt composition for each segment demonstrated that there was a significant difference between human and avian sets in terms of the (d)nts.

**FIg. 2.**
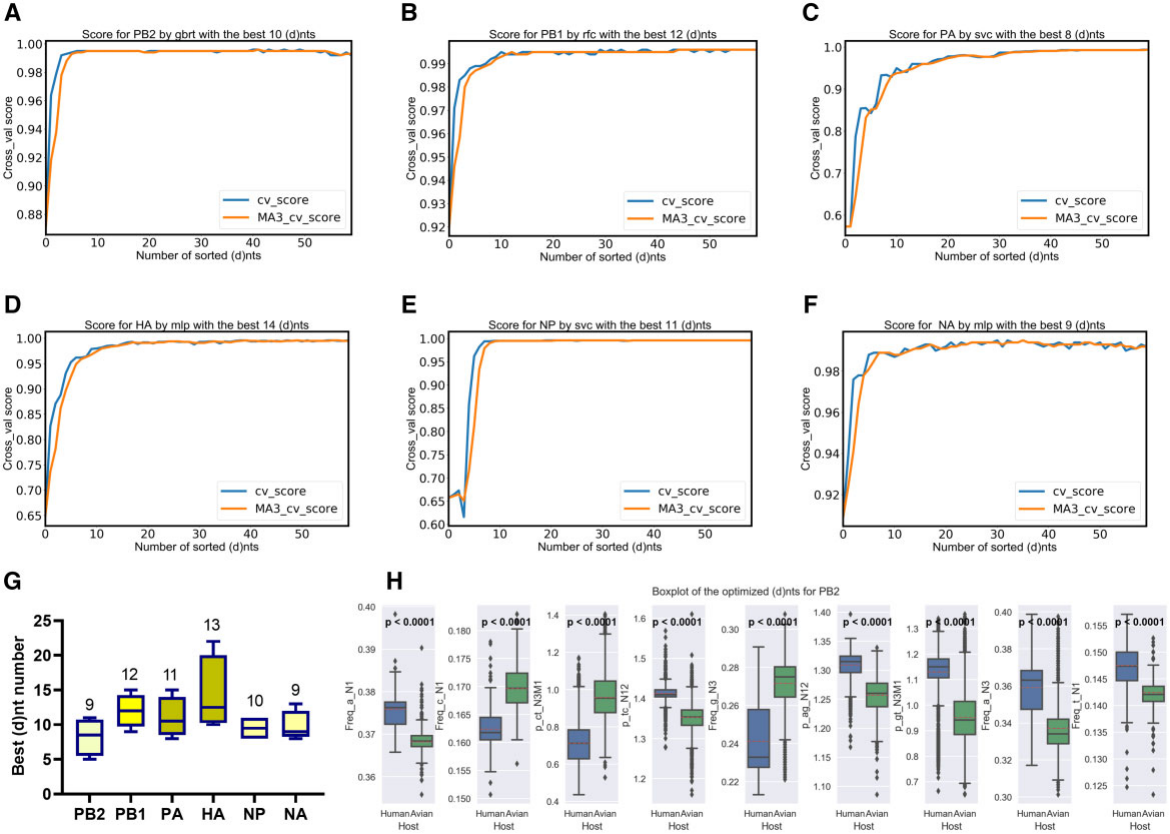
Optimization by SVC with the most different (d)nts between human and avian virus segments. The sorted 60 (d)nts were successively put into the accumulating feature list for the SVC (random state = 1 and cv = 5); the MA of the three cross-validation scores (cross_val importance) (MA3) and the cross_val importance itself for each segment were curved (*A*–*F*). The first 9, 12, 11, 13, 10, 9 (d)nts in each list were defined as the best features for PB2, PB1, PA, HA, NP, and NA (*G*), respectively, when the cross_val importance curve crossed the MA3 curve. (*H*) Boxplot of the best 9 (d)nts for the PB2 segment; the Mann–Whitney *U* test was performed between the two groups for each (d)nt, and the *P*-value is indicated.

The ML-based GBRT, MLP classifier, RF classifier, and SVC models were utilized to identify the optimal number of (d)nts for the human/avian IAV classification. As indicated in figure 2*A*–*F*, a leveling off of the crossing of the cross-validation score (Cross_val score) with its moving average 3 (MA3) level, along with the (d)nt accumulation, was defined as the indicator of the optimal number of (d)nts. Accordingly, an average of 9–13 top (d)nts in the sorted list was identified as the best/optimized (d)nts by the four types of ML classifiers (fig. 2*G*). For the PB2 segment, there was a significant (*P* < 0.001, Mann–Whitney *U* test, supplementary table 8, Supplementary Material online) difference between avian and human sets for each of the nine optimized (d)nts (fig. 2*H*, supplementary fig. 8*A*, Supplementary Material online). A significant (*P* = 0.049461 for p_ag_N12 in the HA segment, *P* < 0.001 for the others, Mann–Whitney *U* test, supplementary table 8, Supplementary Material online) difference was also observed for each of the other (d)nts for the PB1 (supplementary fig. 8*B*, Supplementary Material online), PA (supplementary fig. 8*C*, Supplementary Material online), HA (supplementary fig. 8*D*, Supplementary Material online), NP (supplementary fig. 8*E*, Supplementary Material online), and NA (supplementary fig. 8*F*, Supplementary Material online) segments.

### Predicting Human Adaptation of IAVs Based on the Characterized Nucleotide Composition

**FIg. 3.**
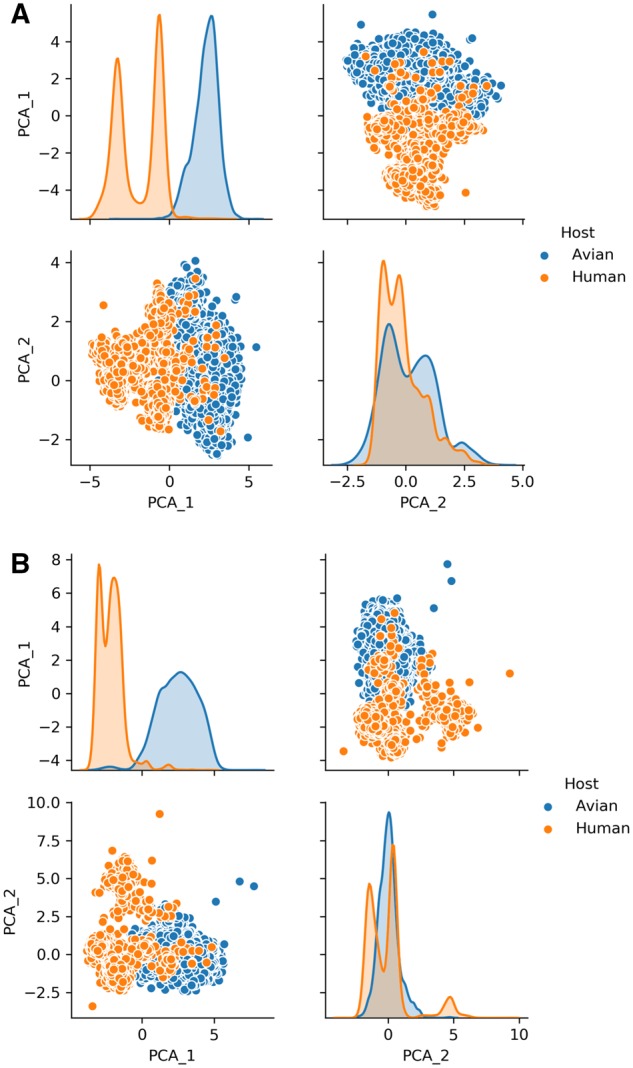
PCA analysis of the optimized (d)nts for the PB2 and PB1 segments between human and avian IAV sequences. The optimized 9 and 12 (d)nts for the PB2 (*A*) and PB1 (*B*) segments, respectively, were converted into two principal components and were then plotted with pairplot (seaborn package, Python) (lower-left panel and upper-right panel in each figure subpart). The distribution of principal components 1 (PCA_1) and 2 (PCA_2) of avian (blue) and human (orange) sequences were indicated by kernel density estimation (KDE) (upper-left panel and lower-right panel in each figure subpart), and the separability between avian and human sequences was shown for the PB2 (*A*) and PB1 (*B*) segments with the pair plots and KDE.

**FIg. 4.**
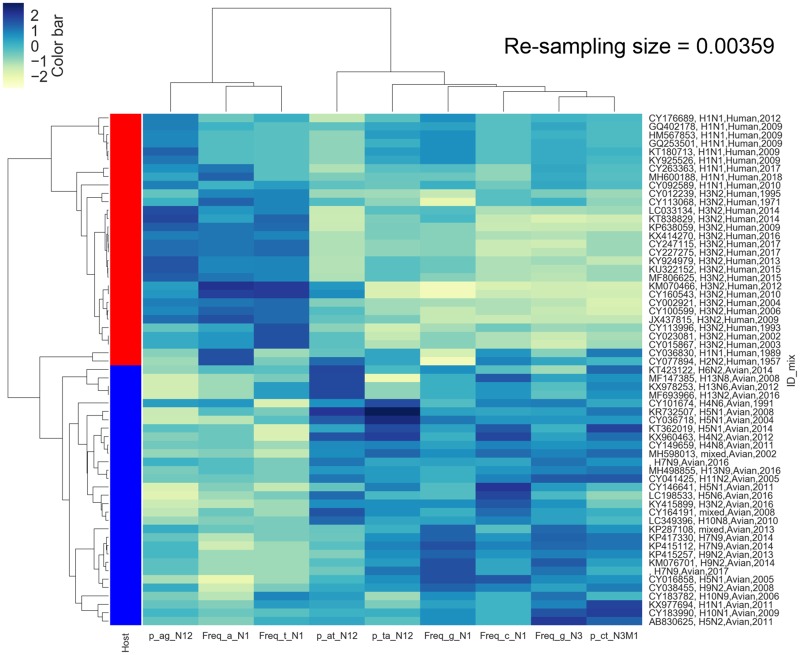
Heatmap and hierarchical clustering of human and avian IAV PB2 sequences based on the Euclidean distance of the optimized (d)nts. The 61 PB2 sequence samples were randomly (random state = 1) selected from PB2 (3.59‰) and then clustered with a heatmap and hierarchical clustering for PB2 based on the Euclidean distance of the optimized 9 (d)nts; the sequence identity and (d)nts were clustered. Standardized scaling was performed for data with the function (x-x.mean)/x.std. The color in the heatmap presented the value for each (d)nt on the *x*-axis, as shown by the color bar in the upper-left corner. The hierarchical relationships for the sampled sequences and (d)nts are indicated on the left and upper sides, respectively, in each image. The red-blue column to the left of the heatmap was utilized to show the human (red) and avian (blue) groups.

The unsupervised clustering and supervised two-category classification with the four ML classifiers mentioned above were performed to evaluate the effectiveness of the characterized (d)nts for human/avian IAV classification. It was demonstrated that the two principal components of the nine optimized (d)nts were separable in distribution between avian and human sequence sets for the PB2 (fig. 3*A*) and PB1 (fig. 3*B*) segments. Such separability was also observed for the PA (supplementary fig. 9*A*, Supplementary Material online), HA (supplementary fig. 9*A*, Supplementary Material online), NP (supplementary fig. 10*A*, Supplementary Material online), and NA segments to varying degrees. Such separability was also indicated by the hierarchical clustering of both sequence sets. The majority of the human and avian PB2 sequences were clustered into two groups for the PB2 segment (fig. 4) and the other five segments (supplementary figs. 11–15, Supplementary Material online), particularly for the ribonucleoprotein complex of the PB2, PB1, and NP segments. Interestingly, the PA sequences of A(H1N1)pdm09 were clustered into the avian sequence group, thought distinctive from other human sequences and avian sequences, as indicated previously (Smith et al. 2009).

**FIg. 5.**
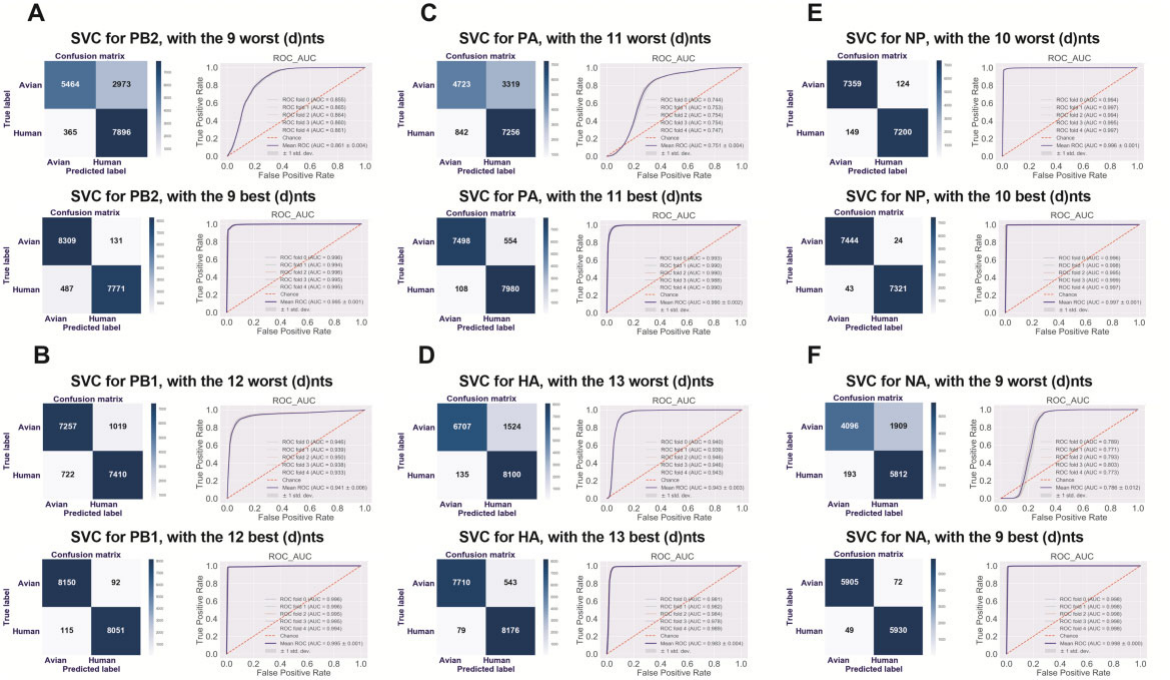
The prediction of human adaptation classes (true/false) and the human adaptation probability by the SVC model, with optimized (d)nts for each segment. The human adaptation classes (true/false) and the human adaptation probability of avian and human sequences were predicted by the SVC with the optimized (best) 9–13 (d)nts for the six segments, with the same optimized-(d)nt number tail (worst) (d)nts as the control. The confusion matrix for human adaptation class prediction, the ROC curve and the area under the ROC curve (AUC) for the SVC model with the worst or best (d)nts are indicated, respectively, for PB2 (*A*), PB1 (*B*), PA (*C*), HA (*D*), NP (*E*), and NA (*F*).

An SVC was used to predict the human adaptation of all IAV sequence samples, with the optimized (d)nts, with the same number of tail (d)nts in the sorted list as control. The true negative rate and the true positive rate for the control (d)nts were 64.76% and 95.58%, respectively, for the PB2 segment (upper-left panel, fig. 5*A*); their average AUC for the 5-fold tests was 0.861 ± 0.004 (upper-right panel, fig. 5*A*). However, the true negative/positive rates for the optimized (d)nts for the PB2 segment were 98.45% and 94.10%, respectively (lower-left panel, fig. 5*A*), and the average AUC increased to 0.995 ± 0.001 (lower-right panel, fig. 5*A*). As indicated in figure 5*B*–*F*, the prediction and the probability of the optimized (d)nt-based SVC was markedly higher than that of the control (d)nt-based SVC. High performance with the optimized (d)nts was also obtained with the other three supervised learning models (GBRT, RF classifier, and MLP classifier) (supplementary Figs. 16–18, Supplementary Material online, respectively).

**FIg. 6.**
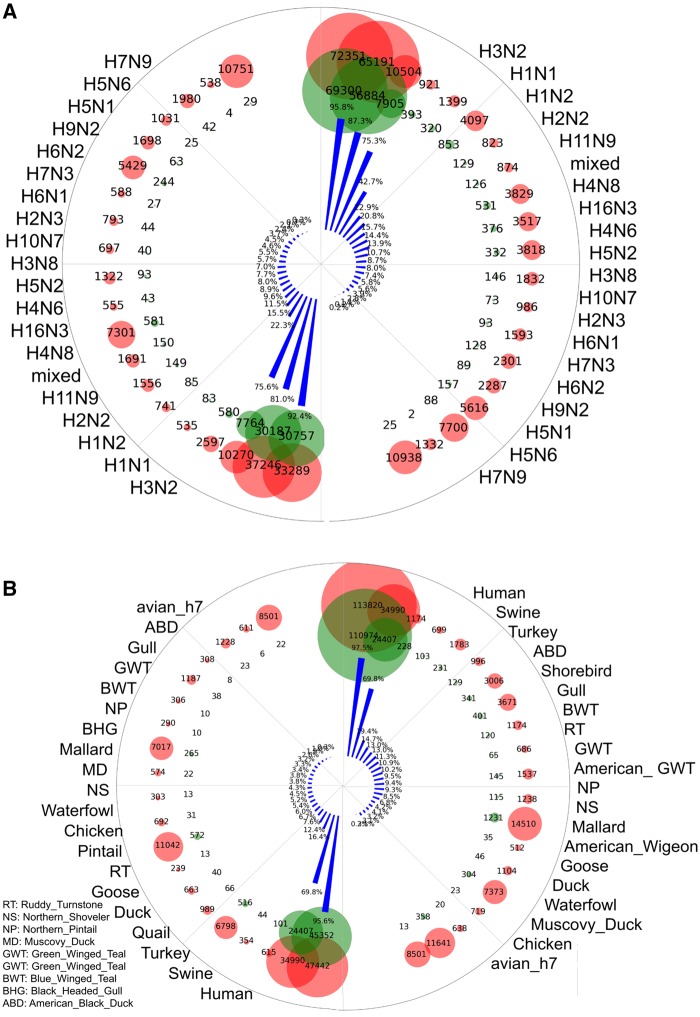
The distribution of the subtypes and hosts of human-adaptive IAV sequences before the 2009 influenza pandemic. The human adaptation was predicted by the SVC model with a probability threshold of 0.5. The total number of sequences, the number of human-adaptive sequences, and the human adaptation ratio are quantitatively presented by the size of the red and green circles and the height of the blue histogram in polar coordinates. The labels of the top 20 subtypes (*A*) or the top 20 hosts (*B*) for the US-resampled sequences (left part of A/B) or for all IAV sequences (right part of A/B) are indicated. The subtypes and hosts are listed in descending order.

The human adaptation of the sequence-resampled sequence set from the United States and all-sequence set was predicted (with an SVC probability threshold of 0.5) by the above-mentioned SVC model. Then, the association of such adaptation was analyzed with sequence labels, such as subtype and host. Regardless of the other labels, the H1N2 subtype was highly adaptive to humans (approximately 75% by both sequence sets, the left and right parts in fig. 6*A*), as well as the designated human-adaptive H3N2 and H1N1 subtypes. There were 10% or more human-adaptive sequences for H2N2 and H11N9, mixed or H4N8 sequences in the sequence-resampled sequence set from the United States (left part of fig. 6*A*), and H16N3 and H4N6 in the all-sequence set (right part of fig. 6*A*). In terms of the host, 4.4% or 2.5% human sequences, mainly from human-infected AIVs, were not adaptive to humans (fig. 6*B*), and almost 70% of swine sequences were human-adaptive sets (fig. 6*B*). Surprisingly, 16.4% and 19.4% of the turkey sequences from the two sets were human-adaptive sets, ranking first for avian hosts; more than 10% sequences were human-adaptive sets from other birds, such as American black ducks, shorebirds, gulls, blue-winged teal, and quails in either sequence set (fig. 6*B*).

**FIg. 7.**
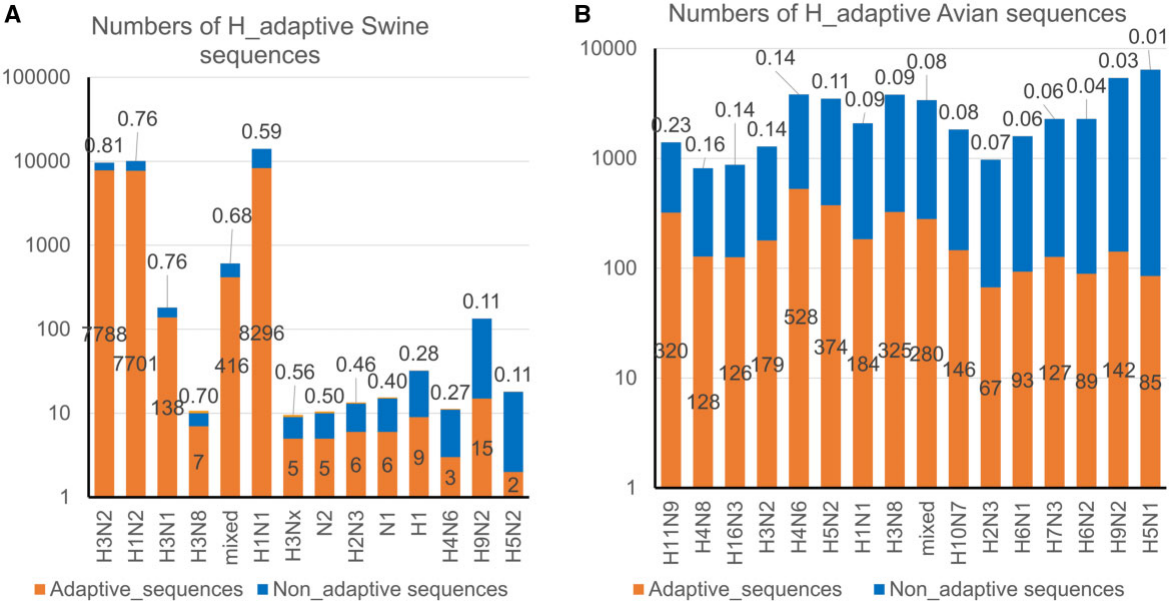
The prediction of the human adaptation of swine and avian IAV sequences before the 2009 influenza pandemic. Human-adaptive swine (*A*) and avian (*B*) IAV sequences before the 2009 influenza pandemic were predicted by the SVC model with a probability threshold of 0.5. The total number of sequences, the number of adaptive sequences, and the human adaptation ratio are represented by the orange, blue, and yellow histograms, respectively. The human adaptation ratio is also presented as a floating number above the stacked histogram. IAV subtypes are indicated by the labels along the *x*-axis.

Additionally, when both the segment and subtype labels were taken into account, more different details appeared in such adaptations. As indicated in figure 7*A*, most of the human-adaptive sequences (more than 50%) from swine were H3 or H1 in the hemagglutinin subtype and were N1 or N2 in neuraminidase subtype, with H3N8 as an exception. For avian sequences, H11N9, H4N8, H16N3, H3N2, H4N6, H5N2, H1N1, and H3N8 were at the top of the list (fig. 7*B*).

### Human Adaptation of Avian and Swine IAVs before and after the 2009 H1N1 Influenza Pandemic

A(H1N1)pdm09 viruses, also known as swine-origin IAVs (H1N1) (S-OIVs), first emerged in North America (Smith et al. 2009) and spread all over the world within the following 6 months (Swerdlow et al. 2011; Fineberg 2014). Origins and evolutionary genomics of S-OIVs have been well identified (Garten et al. 2009; Smith et al. 2009). Given the high importance in influenza pandemics of swine as a mixer for human and avian IAVs (Vijaykrishna et al. 2011; Nelson et al. 2015), we analyzed the worldwide distribution of human-adaptive swine and avian sequences before the 2009 H1N1 influenza pandemic. It was indicated that North America and East Asia were the high-risk areas of human-adaptive swine sequences (table 1) for the six IAV segments, based on the human adaptation ratio. Notably, the United States is ranked first for the absolute number of human-adaptive sequences for each segment. East Asia, particularly Hong Kong and mainland China, had larger human adaptation ratios. Human-adaptive avian sequences are dominantly distributed in the United States and China (table 1). Interestingly, the United States still led the world in the number or proportion of human-adaptive sequences for the five segments (table 1), except NA (maximum in China) (table 1). Accordingly, such distribution bias of human-adaptive swine and avian sequences was consistent with the origin of each segment for the 2009 H1N1 pandemic (Smith et al. 2009).

**Table 1.** Human Adaptation for Swine and Avian IAV Sequences before the 2009 H1N1 Influenza Pandemic in Different Countries/Areas.

Host	Country/Area, Adaptation ratio (%) (Number of adaptive/total sequences)											
	PB2		PB1		PA		HA		NP		NA	
Swine	**JP**	**100.0 (20/20)**	**US**	**99.2 (397/400)**	**JP**	**100.0 (19/19)**	**DK**	**100.0 (21/21)**	**JP**	**100.0 (20/20)**	**DK**	**84.2 (32/38)**
	**HK**	**69.7 (85/122)**	**JP**	**94.7 (18/19)**	**UK**	**96.9 (62/64)**	**UK**	**97.2 (69/71)**	**US**	**97.3 (427/439)**	**KR**	**68.8 (22/32)**
	**CN**	**57.7 (45/78)**	**CA**	**91.7 (44/48)**	**IT**	**94.7 (18/19)**	**JP**	**93.1 (27/29)**	**CA**	**91.7 (44/48)**	**IT**	**57.5 (23/40)**
	US	32.6 (125/384)	**KR**	**82.1 (23/28)**	**HK**	**91.9 (136/148)**	**IT**	**90.5 (38/42)**	**KR**	**83.3 (20/24)**	**CA**	**55.3 (26/47)**
	CA	31.1 (14/45)	**HK**	**78.1 (121/155)**	**TH**	**77.8 (7/9)**	**DE**	**87.8 (36/41)**	**HK**	**78.6 (125/159)**	**UK**	**53.0 (35/66)**
	KR	19.4 (6/31)	**CN**	**60.0 (48/80)**	**CN**	**66.7 (42/63)**	**CA**	**78.0 (39/50)**	**CN**	**60.8 (59/97)**	JP	42.9 (12/28)
	UK	11.1 (7/63)	UK	9.2 (6/65)	**KR**	**59.3 (16/27)**	**HK**	**60.2 (100/166)**	IT	19.4 (6/31)	US	36.6 (158/432)
	IT	7.4 (2/27)	IT	7.4 (2/27)	**DE**	**52.6 (10/19)**	**CN**	**59.6 (68/114)**	UK	12.1 (8/66)	DE	32.6 (14/43)
	FR	6.2 (1/16)	ES	0.0 (0/16)	US	48.6 (192/395)	**US**	**59.2 (274/463)**	FR	5.9 (1/17)	CN	27.2 (25/92)
	DE	0.0 (0/25^b^)	DE	0.0 (0/33)	CA	42.2 (19/45)	TH	48.4 (15/31)	DE	0.0 (0/30)	HK	16.8 (46/274)
Avian	**US**	**8.4 (190/2,266)**	CA	3.5 (17/479)	**US**	**33.2 (758/2,286)**	**AUS**	**51.2 (22/43)**	US	1.7 (33/1,935)	**JP**	**7.0 (3/43)**
	**CN**	**5.0 (27/542)**	US	2.2 (51/2,282)	**CA**	**28.9 (129/446)**	**DE**	**36.6 (15/41)**	HK	1.6 (1/63)	**CN**	**5.7 (23/407)**
	CA	3.4 (16/474)	CN	0.4 (2/534)	**NL**	**26.7 (58/217)**	**RU**	**19.5 (8/41)**	CA	0.2 (1/417)	TH	3.0 (1/33)
	HK	3.2 (2/62)	VN	0.0 (0/89)	**AUS**	**17.0 (8/47)**	**CA**	**18.2 (87/478)**	IT	0.0 (0/79)	TW	2.6 (1/39)
	JP	3.1 (2/65)	IT	0.0 (0/91)	**TW**	**8.1 (3/37)**	**US**	**17.5 (352/2,008)**	VN	0.0 (0/92)	HK	1.9 (1/52)
	NL	0.4 (1/225)	NL	0.0 (0/219)	**CN**	**6.8 (30/440)**	**JP**	**14.3 (10/70)**	NL	0.0 (0/209)	US	1.7 (17/976)
	AUS	0.0 (0/47)	SE	0.0 (0/237)	**DE**	**6.2 (2/32)**	**NL**	**13.4 (28/209)**	SE	0.0 (0/232)	SE	1.6 (1/62)
	RU	0.0 (0/47)	\	\	**IL**	**5.4 (2/37)**	**HK**	**9.4 (6/64)**	CN	0.0 (0/522)	NL	0.9 (1/106)
	TH	0.0 (0/54)	\	\	JP	4.8 (3/63)	**SE**	**8.5 (20/236)**	\	\	CA	0.5 (1/184)
	IT	0.0 (0/82)	\	\	HK	3.1 (2/64)	**TW**	**7.7 (3/39)**	\	\	IL	0.0 (0/36)

Countries/areas were abbreviated as Australia: AUS, Canada: CA, China: CN, Spain: ES, France: FR, Germany: DE, Denmark: DK, Hong Kong, China: HK, Israel: IL, Italy: IT, Japan: JP, South Korea: KR, Netherlands: NL, Russia: RU, Sweden: SE, Taiwan, China: TW, Thailand: TH, United Kingdom: UK, USA: US, Vietnam: VN.

Furthermore, the hierarchical clustering analysis of the S-OIVs with the IAVs before 2009 was performed based on the SVC model-characterized (d)nts by random sampling 1,000 samples from the total samples. As indicated (supplementary fig. 19*A*, Supplementary Material online), the S-OIV (A(H1N1)pdm09) PB2 sequences were clustered most closely with the PB2 sequences from several avian viruses (H6N8, H11N2, H11N9, and mixed subtypes) in Delaware in 1994 and 1995 and then with the avian and swine viruses (H1N1, H3N2, and H5N2) in Delaware and other US states in the 2000s. S-OIV PB1 was most closely clustered with the human H3N2 viruses before or after 2000 in the United States (supplementary fig. 19*B*, Supplementary Material online). The close clustering with various subtypes of avian/swine viruses mainly in the United States and sporadically in East Asia for the PA segment (supplementary fig. 19*C*, Supplementary Material online) and the neighboring with swine H1N1, H1N2, and H3N2 viruses in the United States /Asia/Europe for the HA, NP, and NA segments were indicated (supplementary fig. 19*D*–*F*, Supplementary Material online). Such kinds of clustering for the six segments were also indicated by the other nine rounds of hierarchical clustering analysis with random-resampled sequences (random state = 2–10) (supporting data for supplementary fig. 19, Supplementary Material online). Interestingly, the results were consistent with a previous evolutionary analysis with full cDNA sequences (Smith et al. 2009). Moreover, as indicated by the human adaptation probability in each clustering hierarchy (the last floating number in each sequence name, supplementary fig. 19, Supplementary Material online), almost all of the A(H1N1)pdm09-neighboring avian or swine sequences were predicted by our model to be human-adaptive sequences.

The influence of the 2009 H1N1 influenza pandemic on the human adaptation of avian and swine IAVs post-2009 was also evaluated. As indicated in table 2, all six segments of avian sequences decreased in terms of the human adaptation ratio, although the adaptive sequence number increased. In particular, the adaptive sequences of PB1, PA, and HA increased much more (over the median level). For swine viruses, all six segments of the sequences, particularly for NA, PA, and HA (over the median level), increased in terms of both the human adaptation ratio and adaptive sequence number.

**Table 2.** Human Adaptation Changes in Avian, Swine, and Human IAVs after the 2009 H1N1 Influenza Pandemic.

Segment	Period/Change	Adaptation Ratio (%) (adaptive/total sequences) and the Change (%)		
		Avian	Swine	Human
PB2	Before 2009	5.4 (249/4,608)	35.6 (311/873)	87.3 (3,397/3,893)
	2009–2018	2.6 (334/12,846)	35.9 (1,574/4,379)	95.6 (16,415/17,179)
	Change	−51.85	0.84	9.51
PB1	Before 2009	**1.91 (88/4,610)**	71.2 (664/933)	93.2 (3,627/3,890)
	2009–2018	**1.87 (236/12,598)**	84.5 (3,734/4,421)	97.6 (16,456/16,856)
	Change	−**2.11**	18.68	4.72
PA	Before 2009	**22.7 (1,019/4,487)**	**64.7 (549/849)**	95.9 (3,633/3,790)
	2009–2018	**19.0 (2,351/12,398)**	**84.2 (3,508/4,168)**	98.2 (16,550/16,856)
	Change	−**16.30**	**30.14**	2.40
HA	Before 2009	**14.0 (604/4,314)**	**66.7 (753/1,129)**	94.5 (4,809/5,091)
	2009–2018	**11.5 (1,398/12,158)**	**79.4 (7,234/9,113)**	97.7 (27,508/28,158)
	Change	−**17.86**	**19.04**	3.39
NP	Before 2009	1.1 (47/4,133)	72 (717/996)	95.7 (3,517/3,676)
	2009–2018	0.6 (65/11,005)	83.8 (3,709/4,427)	98 (14,101/14,385)
	Change	−45.45	16.39	2.40
NA	Before 2009	2.4 (58/2,379)	**35.8 (429/1,200)**	93.6 (3,544/3,787)
	2009–2018	1.4 (109/7,734)	**54.8 (4,648/8,482)**	97.8 (19,944/20,386)
	Change	−41.67	**53.07**	4.49

## Discussion

IAVs must acquire sufficient human adaptation before they can promote human pandemics. To date, there has been no universal definition of IAVs’ human adaptation, although numerous human-adaptive viral determinants have been reported (Taubenberger and Kash 2010; Bouvier 2015; Long et al. 2019). In the present study, we defined it as the capability to infect humans easily, to transmit among populations efficiently, and to be virulent to some degree to humans. Accordingly, the human-adaptive IAVs were limited to the H3N2 and H1N1 viruses, either of which can continuously cause endemics or even pandemics in humans (Ren et al. 2016), whereas other subtypes of avian IAVs (Yang et al. 2007; Rudge and Coker 2013; Hu et al. 2014) were classified into the avian-adaptive group. There might be a concern about a selection bias of the human adaptation criteria. If so, the “human adaptation” label for the four segments (PB2, PB1, PA, and NP) would be wrongly associated with “H3N2” or “H1N1,” which would be inconsistent with the “true” human adaptation of these segments. Under such circumstances, the “true” human-adapted PB2, PB1, PA, and NP sequences of the human-adaptive H2N2 virus (Cox and Subbarao 2000) might be underestimated, and the host adaptation of the four segments of swine H1N2 virus would not be correctly predicted. However, high HA and NA of the human adaptation frequencies were unanimously predicted for the PB2, PB1, PA, and NP sequences for both the H2N2 and H1N2 viruses. Interestingly, since 2005, dozens of human H1N2 infection cases have been reported in the United States (Pulit-Penaloza et al. 2018) and the Netherlands (Meijer et al. 2018); the high human adaptation of the H1N2 virus was also experimentally supported (Pulit-Penaloza et al. 2018). Therefore, the IAV human adaptation criteria are acceptable to some degree, according to the existing human-adapted IAVs.

In the last few decades, the overwhelming majority of studies on viral determinants have focused on protein levels for virus infection, transmission, virulence, and host adaptation. In particular, distinct protein signatures for host tropism (Eng et al. 2016) and avian-to-human transmission (Qiang et al. 2018) have been recognized with ML methods. Recently, accumulating reports found a significant influence of synonymous viral nucleotide or dinucleotide mutation on the virus response to the host’s innate immune system (Takata et al. 2017) on virus virulence (Atkinson et al. 2014; Tulloch et al. 2014) and virus replication (Witteveldt, Martin-Gans and Simmonds 2016). Here, we compressed the full-length coding information of the six segments into the counting information of 12 nts and 48 dnts, all of which were sorted according to their classification importance with the PCA/SVC method. Optimization is one of the crucial parts of machine/deep learning. A moving average (MA; Kashyap 1982), also known as the rolling mean, was utilized here to optimize the number of features for the ML models, with the crossing of the MA with its MA3 value as a cutoff point, at which the number of (d)nts was the best.

Interestingly, the counting information of each mono- or dinucleotide varied in the importance of each segment. Besides, 9–13 optimized (d)nts were enough to predict the human adaptation for each of the six segments. Given the high performance in the avian/human adaptation classification, no other optimization methods were explored here.

The species-specific (Glass et al. 2007) and virus-family-specific (Di Giallonardo et al. 2017) dinucleotide composition has been computationally explored for viruses. The genomic dinucleotide composition of RNA viruses is useful for predicting viral reservoir hosts and arthropod vectors (Babayan et al. 2018). Therefore, we speculated here that the mono-/dinucleotide composition should be another critical genomic feature, and we assume here that there should be a species selection bias of IAV nucleotides/dinucleotides. Taking PB2 as an example, the frequency of T, C, A, or G at the first position and G at the third position within a codon, the odds ratios of ct_N3M1, ag_N12 and at_N12 determined the human adaptation of IAVs. According to the eukaryotic codon list (Shu 2017), every amino acid is coded by one (for methionine and tryptophan) to six trinucleotide codons (for leucine, serine, and arginine); six (phenylalanine, leucine, serine, tyrosine, cysteine, and tryptophan), five, seven, and five types of amino acids were respectively dependent on the nucleotides of T, C, A, and G, at the first nucleotide position within a codon, 4–7 types of amino acids were dependent on the four types of nucleotides at the first nucleotide position, and 13–15 types of amino acids were dependent on the four types of nucleotides at the third nucleotide position. Therefore, each mononucleotide feature is theoretically associated only with 5–15 possible amino acids (the stop codon is not considered). Accordingly, every dinucleotide is associated with 1–4 types of amino acids (Shu 2017). Therefore, the nucleotide composition was associated only with the amino acid compositional information, with less than 50% probability. Thus, we speculated that the genomic composition of mono-/dinucleotides is another essential genomic characteristic of IAVs and is probably biologically associated with the host adaptation of IAVs.

An A(H1N1)pdm09 virus caused the latest worldwide influenza pandemic (Swerdlow et al. 2011; Fineberg 2014). Here, our results regarding the high human adaptability of swine IAVs before 2009 in the United States precisely predicted the high risk of these IAVs. Of course, a possible underestimation of human-adaptive swine viruses/sequences was not excluded in many high-risk developing countries, such as China and Vietnam, due to a likely undeveloped monitoring/detection program for swine influenza. However, a marked lower human adaptation of the swine IAV sequence was also indicated by our model in the area of the European Union, which is the second largest pig plantation area, with twice as much pig production in this area in 2018 than the United States (https://www.statista.com/statistics/273232/net-pork-production-worldwide-by-country/). Moreover, the 2009 H1N1 pandemic was not initiated in China, although pork production in China is 4-fold that of the United States. This phenomenon implies that our results might reveal the “true” severity of swine infection of human-adaptive swine viruses in the United States rather than in other areas before 2009.

In summary, the human-adaptive and avian-adaptive nucleotide compositions of influenza A viruses (IAVs) were determined with supervised/unsupervised ML methods. ML, based on human-adaptive nucleotide composition, performed well in predicting the human adaptation of IAVs before the 2009 H1N1 pandemic. This approach might be promising for the prediction of the risk of an influenza pandemic and global vulnerability to influenza.

## Materials and Methods

### Sequence Data Processing

Full-length coding sequences of the first six IAV segments of PB2, PB1, PA, HA, NP, and NA (the M and NS segments are not included due to their short length) were utilized for the nucleotide composition analysis. In total, 115,917 human sequence samples, 76,538 avian sequences, and 35,569 swine sequences, up to December 31, 2018, were downloaded from the Influenza Research Database (IRD) (Zhang et al. 2017) or from the Global Initiative on Sharing All Influenza Data (GISAID) database (Shu and McCauley 2017). The ID, strain name, sequence length, and other labels were extracted from the definition content of sequence file in FASTA format via a Python script (Script-1, supplementary data, Supplementary Material online). The ID number and other labels are listed in the supplementary data, Supplementary Material online. The composition of mononucleotide (nt, T, C, A, and G) and dinucleotides (dnts, 16 types of combination of every two nts) were counted and calculated according to formulas 1 and 2/3 (Script-2, supplementary file, Supplementary Material online) for each of the three types of nucleotide positions within a trinucleotide codon (Karlin and Mrazek 1997). In total, there were 60 (d)nts, including the frequency of 12 types of nts ($\mathrm{fr}eqx_{n}$) and the relative frequency of 48 types of dnts ($\rho_{x_{n}y_{n}}$).

(1) $$\mathrm{fr}eqx_{n}=\frac{\Sigma x_{n}}{\sum_{i=1}^{4}x_{n}},\;{(x}_{n}=T,\;C,\;A\;\mathrm{or}\;G,\;n=\mathrm{codon}\;\mathrm{nt}\;\mathrm{position}\;1,\;2,\;\mathrm{or}\;3)$$

(2) $$\mathrm{fr}eq{x_{n}y}_{m}=\frac{\Sigma x_{n}y_{m}}{\sum_{i=1}^{16}{x_{n}y}_{m}},\;(x,\;y=\;T,\;C,\;A\;\mathrm{or}\;G,\;m=n+1\;\mathrm{for}\;m\leq 3,\;m=n-2\;\mathrm{for}\;m=4,\;n=\;\mathrm{codon}\;\mathrm{nt}\;\mathrm{position}\;1,\;2,\;\mathrm{or}\;3)$$

(3) $$\rho_{x_{n}y_{n}}=\frac{\mathrm{fr}eqx_{n}y_{m}}{\mathrm{fr}eqx_{n}*\mathrm{fr}eqy_{m}},\;{(x}_{n\;}{,y}_{m\;}=T,\;C,\;A\;\mathrm{or}\;G,\;n=\mathrm{codon}\;\mathrm{nt}\;\mathrm{position}\;1,\;2,\;\mathrm{or}\;3,\;m=n+1\;\mathrm{for}\;m\leq 3,\;m=n-2\;\mathrm{for}\;m=4)$$

Sequences with duplicate ID, incorrect labels, or out-of-length ranges were excluded, and the remaining 217,549 sequences are listed in supplementary table 1, Supplementary Material online. To avoid a country/area bias for ML modeling, due to the overwhelming majority of US samples, we randomly resampled the US sequences, with the United States to China ratio of approximately 1:1 for each segment (Script-3, supplementary file, Supplementary Material online), via the pandas.DataFrame.sample (Python) model. In total, 46,042 randomly resampled human-adaptive sequences and 46,488 human-inadaptive avian sequences were utilized for feature extraction and model building. Human-originated H5N1, H7N9, and other subtypes were excluded from the human adaptation set and were not included in the avian set for model building.

### The Phylogenetic Analysis of Randomly Sampled Human/Avian IAV Sequences Using the Maximum Likelihood Method

In total, 59–61 sequence samples were randomly selected (random state = 1) from each segment sequence set (3.59–5.01% of the total sequences) via pandas.DataFrame.sample (Python). Then, MEGA (MEGA 7.0.26, Kumar et al. 2016) was utilized to build a maximum likelihood tree with the Tamura–Nei model (Tamura and Nei 1993) for PB2 (A) and the other five segments (B–F). The parameters were set as follows: uniform rates among sites, gaps complete deletion, the ML heuristic method set to the nearest-neighbor interchange, and with making the initial tree automatically (default—NJ/BioNJ) as an initial tree. Multiple and pairwise alignments were performed with ClustalW with a gap-opening penalty of 15, a gap extension penalty of 6.66, an IUB DNA weight matrix, and a transition weight of 0.5 before a phylogenetic tree was built. Another two rounds of random resampling were performed from each segment sequence set with the same sampling ratio as mentioned above, and the maximum likelihood tree was built with the same parameters.

### Machine Learning Analysis

ML analysis was performed with Python. The Scikit-learn package (version = 0.18.1, https://scikit-learn.org/stable/#) was utilized for PCA (sklearn.decomposition.PCA) analysis and the supervised ML methods of SVC, GBRT, MLP classifier, and RF classifier from the submodel of sklearn.svm.SVC, sklearn.ensemble.GradientBoostingClassifier, sklearn.neural_network.MLPClassifier, and sklearn.ensemble.RandomForestClassifier, respectively. The data were split with StratifiedKFold from sklearn.model_selection (n_splits = 5, random_state = 1, shuffle = True) into five training/test sets before supervised learning was implemented (Script-4, supplementary file, Supplementary Material online). The SciPy package (cluster.hierarchy, version = 0.19.0, https://www.scipy.org) was utilized to build a hierarchical clustering of the IAV sequences based on the Euclidean distance between/among sequences.

PCA is a widely utilized unsupervised ML model for constructing a low-rank model of a data matrix. For the following (d)nt sorting, an orthogonal transformation by PCA (Jolliffe and Cadima 2016) was performed to convert every four (d)nts with possible correlations into one principal component, with the most significant possible variance (formula 4). For the evaluation of the separability between avian and human sequences, PCA was also utilized to transform the information of all (60) or the optimized (d)nts into two principal components.

Hierarchical clustering is another important unsupervised ML method for hierarchical cluster analysis. Strategies for hierarchical clustering generally fall into two types: “bottom-up” approaches, by which each observation starts from a lower cluster and then are clustered with its paired cluster(s) in a higher hierarchy; and “top-down” approaches, by which all the observations start from the top cluster and then are split into lower hierarchies recursively down the hierarchy axis. For the hierarchical clustering of the IAV sequences based on all of or the optimized (d)nts, the Euclidean distance was calculated and utilized as a hierarchical clustering scalar (formula 5).

(4) $$\mathrm{minimize}\;{\left\|A-\mathrm{XY}\right\|}_{F}^{2}=\sum_{i=1}^{m}\{\sum_{j=1}^{n}{\left(A_{\mathrm{ij}}-x_{i}y_{j}\right)}^{2}\},\;s.t.\;\;X\epsilon R^{m\times k},\;Y\epsilon R^{k\times n},\;k<m\;\mathrm{or}\;n$$

(5) $${\left\|a-b\right\|}_{2}=\sqrt{\;\sum_{i=1}^{n}{\left(a_{i}-b_{i}\right)}^{2}},\;a,\;b=\mathrm{avian},\;\mathrm{human}\;d\left(\mathrm{nt}\right);\;1\leq \;n\leq \;60$$

An SVC, also known as a support vector machine or a support vector network (Noble 2006), is one of the most popular supervised learning models for classification and regression analysis. An SVM training algorithm builds a model that assigns new samples to one category or another, making it a nonprobabilistic binary linear classifier (Noble 2006) (formula 6). The other three ML models were the GBRT MLP classifier and RFC classifier algorithms, by which a prediction is made to evaluate the probability of human adaptation for each sequence. The GBRT algorithm, also known as gradient tree boosting, is a greedy generalized boosting model for differentiable loss functions.

(6) $$\underset{w,b}{\min}\frac{1}{2}{\left\|\omega \right\|}^{2},\;s.t.\;\;y_{i}\left(\omega^{T}x_{i}+b\right)\geq 1,i=1,\;2,\;\ldots ,\;60$$

### Feature Extraction

To evaluate the importance of each (d)nt, we first sorted the (d)nts with a PCA/SVC combined model. Three thousand and five hundred and forty iterations of PCA/SVC analysis were performed to transform every four (d)nts into one PCA component, which was then utilized for the SVC classification of the avian and human IAV sequences. Thus, the 60 (d)nts were sorted according to their average area under the curve (AUC) (a) of the repeated above-mentioned PCA/SVC analysis. Supervised ML models (GBRT, MLP, RFC, and SVC) were utilized to evaluate the efficiency of the sorted (d)nts as human/avian classification features. Accumulated (d)nts, from 1 to 60 from the sorted list, were input into each of the four models, and the Cross_val score was utilized as an efficiency indicator. The optimized ML (d)nt number was defined as the number of the accumulated (d)nts, with which the Cross_val score did not increase as much as the (d)nt number accumulation, was evaluated by the MA strategy (Slawnych et al. 2009) and was determined at the crossing point of the Cross_val score curve with its MA3 curve.

## Data Availability

Original sequence data are available from the Influenza Research Database (IRD, up to December 31, 2018) (Zhang et al. 2017) via the website of www.fludb.org and from the Global Initiative on Sharing All Influenza Data (GISAID) (Shu and McCauley 2017) via the website of www.gisad.org. All the original data for the results are available online: https://github.com/Jamalijama/Predict_IAV_Host.

## Code Availability

The source code for the present study is available online: https://github.com/Jamalijama/Predict_IAV_Host.

## Supplementary Material

msz276_Supplementary_Data
