# Supplementary material for: Machine Learning Methods for Predicting Human-Adaptive Influenza A Viruses Based on Viral Nucleotide Compositions
Source: Mol Biol Evol. 2019 Nov 21;37(4):1224–36. doi: 10.1093/molbev/msz276 (PMC7086167; doi:10.1093/molbev/msz276)

Supplemental information

1. **Supplemental information presents detailed information of supplemental results, supplemental Methods, supporting materials and programming scripts for supplemental information.**
2. **Supplemental results include the description about Supplemental tables, the legends of supplemental Figures, Supplemental tables and supporting data.**
3. **Supplemental Methods described in details about Feature Engineering and sequence resampling.**
4. **Supplemental Figures were uploaded as independent files. Supplemental Figure 19 with high resolution was provided at https://github.com/Jamalijama/Predict_IAV_Host.**
5. **Supporting data for figures, supplemental figures and programming scripts were also provided respectively.**

Supplemental results

1. Supplemental Tables

Supplemental table 1. List of the full-length IAV coding sequences within the length range.

Sequence samples with the labels of Host, Subtype and Segment were listed, post the dropout of 8,634 sequences, due to the length range or the repeated sequence IDs. The length range was set as mean ± 3 * std (2280 ± 9, 2274 ± 9, 2151 ± 9, 1695 ± 27, 1497 ± 9 and 1380 ± 33, respectively for PB2, PB1, PA, HA, NP and NA).

Supplemental Table 2-7, cv_score and its rolling mean for ML models for 6 segments.

The cv_score and its rolling mean (moving average) 3 (MA3) were listed for the model of GBRT, MLP, RFC and SVC respectively. For PB1 with MLP model, the second downcross of cv_score with its MA3 (at the 11th feature number) was designated as the threshold. In another word, the best feature number indicated by the MLP model for PB1 was 10. For all models for the six segments, the cv_score and its MA3 value were listed respectively.

2. Legends for supplemental Figures

Supplemental Figure 1. Numbers of the full-length IAV coding sequences from different countries/areas, hosts, subtypes, segments and years.

List of all the full-length influenza A virus (IAV) coding sequences since December 31st, 2018. Samples from different countries/areas (A), hosts (B), subtypes (C), segments (D) and years (E) were counted and presented as histograms. Values were sorted on a descending turn, and the y-axis was set with logarithmic tick for figure subpart A and E, with linear tick for others.

Supplemental Figure 2. Distribution of the IAV sequences, post a random resampling, in the labels of countries/areas, hosts, subtypes, segments and years.

A random resampling was performed to keep an approximate sample ratio of 1:1 for the country of the USA and China. Samples from different countries/areas (A), hosts (B), subtypes (C), segments (D) and years (E) were counted and presented as histograms. Values were sorted with a descending turn, and the y-axis was set with logarithmic tick for figure subpart A and E, with linear tick for others.

Supplemental Figure 3. Heatmap and hierarchical clustering of randomly-sampled human and avian IAV sequences basing on the Euclidean distance of the 60 (d)nts.

59-61 sequence samples were randomly (random state = 1) selected from each segment sequence set (3.59‰ to 5.01‰ of total sequences), and then were clustered with heatmap and hierarchical clustering for PB2 (A) and the other 5 segments (B-F), based on the Euclidean distance of the 48 di-nucleotides and the 12 mono-nucleotides

respectively; Sequence identity and (d)nts were clustered respectively. Standardized scaling was performed for data with the function of (x-x.mean)/x.std. Color in the heatmap presented the value for each (d)nt in x-axis, as showing by the color bar in the left-top. The hierarchical relationships for the sampled sequences and for (d)nts were respectively indicated in the left and top side in each image. The red-blue column in the left of heatmap was utilized to show the human (red) and avian (blue) group.

Supplemental Figure 4. Phylogenetic analysis of randomly-sampled IAV sequences with maximum likelihood method.

59-61 sequence samples were randomly (random state = 1) selected from each segment sequence set (3.59‰ to 5.01‰ of total sequences), and then were utilized for phylogenetic analysis with MEGA software (MEGA 7.0.26), for PB2 (A), PB1 (B), PA (C), HA (D), NP (E) and NA (F). The sequence ID was indicated as segment, subtype and the strain name from left to right, respectively; the slash "/" in strain name was automatically replaced with a blank by MEGA software.

Supplemental Figure 5. PCA analysis of the 60 (d)nts between human and avian IAV sequences

The 48 dnts and the 12 nts for PB2 (A), PB1 (B), PA (C), HA (D), NP (E) or NA (F) were converted into two principal components and then were plotted with pairplot (seaborn package, python) (left-down and right-up in each figure subpart). The distribution of principal component 1 (PCA_1) and 2 (PCA_2) of avian (blue) and human (orange) sequences was indicated by kernel density estimation (KDE) (left-up and right-down in each figure subpart), and the separability between avian and human sequences was shown respectively for the six segments (A-F) , with the pairplot and KDE.

Supplemental Figure 6. Sampling times for each of the 60 (d)nts for the PCA / SVC optimizer for each segment.

Characterization of human adaption-associated nucleotide composition of IAVs from the 60 (d)nts was performed with combined PCA and SVC. Independent performing times for each (d)nt for the six segments (A-F) in the 3540 iterations of PCA/SVC.

Supplemental Figure 7. Sorting of the 60 (d)nts by the PCA / SVC optimizer for each segment.

3540 iterations of PCA/SVC were performed with randomly-selected four of the 60 (d)nts reduced into one component classify avian and human IAV sequences. The importance of each (d)nt was sorted according to their area under curve (AUC) score of PCA/SVC (A-F).

Supplemental Figure 8. Difference in the PCA/SVC-optimized (d)nts between avian and human IAV segment sequences.

The relative levels of the 9-13 optimized (d)nts for avian (A) and human (H) sequences were plotted with boxplot, for PB2 (A), PB1 (B), PA (C), HA (D), NP (E)

and NA (F). The top whisker, the top boarder, the middle line, the bottom boarder and the bottom whisker were respectively presented the maximum value, 75%-, 50%- and 25%- quantile values and the minimum value, and in which outliers were indicated as diamonds.

Supplemental Figure 9. PCA analysis of the optimized (d)nts for PA and HA between human and avian IAV sequences

The optimized 11 and 13 (d)nts for PA (A) and HA (B), respectively, were converted into two principal components and then were plotted with pairplot (seaborn package, python) (left-down and right-up in each figure subpart). The distribution of principal component 1 (PCA_1) and 2 (PCA_2) of avian (blue) and human (orange) sequences was indicated by kernel density estimation (KDE) (left-up and right-down in each figure subpart), and the separability between avian and human sequences was shown respectively for PA (A) and HA (B), with the pairplot and KDE.

Supplemental Figure 10. PCA analysis of the optimized (d)nts for NP and NA between human and avian IAV sequences

The optimized 10 and 9 (d)nts for NP (A) and NA (B), respectively, were converted into two principal components and then were plotted with pairplot (seaborn package, python) (left-down and right-up in each figure subpart). The distribution of principal component 1 (PCA_1) and 2 (PCA_2) of avian (blue) and human (orange) sequences was indicated by kernel density estimation (KDE) (left-up and right-down in each figure subpart), and the separability between avian and human sequences was shown respectively for NP (A) and NA (B), with the pairplot and KDE.

Supplemental Figure 11-15. Heatmap and hierarchical clustering of human and avian IAV sequences basing on the Euclidean distance of the optimized (d)nts.

59-61 sequence samples were randomly (random state = 1) selected from PB1, PA, HA, NP and NA (respectively for **Supplemental** Figure 11-15) and then were clustered with heatmap and hierarchical clustering, based on the Euclidean distance of the optimized 12, 11, 13, 10 and 9 (d)nts, respectively for PB1, PA, HA, NP and NP; Sequence identity and (d)nts were clustered respectively. Standardized scaling was performed for data with the function of (x-x.mean)/x.std. Color in the heatmap presented the value for each (d)nt in x-axis, as showing by the color bar in the left-top. The hierarchical relationships for the sampled sequences and for (d)nts were respectively indicated in the left and top side in each image. The red-blue column in the left of heatmap was utilized to show the human (red) and avian (blue) group.

Supplemental Figure 16-18. The prediction of human adaption classes (True/False) and the human adaption probability by the GBRT, MLP or RFC model, with optimized (d)nts for the six segments.

The human adaption classes (True/False) and the human adaption probability of avian and human sequences were predicted by SVC with the optimized (best) 9, 12, 11, 13, 10 and 9 (d)nts respectively for PB2, PB1, PA, HA, NP and NP, with same optimized-(d)nt number of tail (worst) (d)nts as control, respectively. The confusion

matrix of human adaption class prediction, the Receiver Operating Characteristic (ROC) and Area Under ROC Curve (AUC) for the GBRT (**Supplemental** Figure 16), MLP (**Supplemental** Figure 17) or RFC (**Supplemental** Figure 18), model with the worst or with the best (d)nts were indicated respectively for PB2 (A), PB1 (B), PA (C), HA (D), NP (E) and NA (F).

Supplemental Figure 19. Heatmap and hierarchical clustering of randomly-sampled IAV sequences before with pd09H1N1 sequences basing on the Euclidean distance of the 60 (d)nts.

1000 IAV sequences were randomly-resampled (random state = 1) from the IAV sequences before 2009 for each segment, and then were clustered with the pd09H1N1 sequences by the heatmap and hierarchical clustering methods for PB2 (A), PB1 (B), PA (C), HA (D), NP (E) and NA (F), respectively, based on the Euclidean distance of the optimized 9, 12, 11, 13, 10, 9 (d)nts respectively. H1N1 IAV virus strains isolated on April, 2009 in USA were taken as example sequences. The labels of sequence ID, host, subtype, year, country/area and human-adaption probability were isolated from the sequence name and were indicted as a mixed sequence ID in the Heatmap and hierarchical clustering. Host for all sequences was also indicated as blue (avian or human), green (swine), red (avian or human) and white (pd09H1N1) respectively.

3. **Supplemental tables**

Supplemental table 1. Numbers of the sequence with the label of segment, host and subtype

**Host**	**Subtypes**	**Sequence number for each segment**						**Total**	**Total**
		**PB2**	**PB1**	**PA**	**HA**	**NP**	**NA**		
	H1N1	387	380	354	275	328	363	2,087	
**Avian**	H3N2	223	225	230	198	199	207	1,282	68,739
	Others	12,236	11,993	11,814	11,685	10,478	7,164	65,370	
	H1N1	7,274	7,043	7,379	13,426	5,949	7,960	49,031	
**Human**	H3N2	9,400	9,343	8,975	13,956	7,948	11,826	61,448	113,820
	Others	505	470	502	776	488	600	3,341	
	H1N1	1,746	1,820	1,687	3,579	1,832	3,409	14,073	
**Swine**	H3N2	1,293	1,303	1,212	2,336	1,274	2,203	9,621	34,990
	Others	1,340	1,298	1,269	3,198	1,321	2,870	11,296	
**Total_Avian**	H1N1	12,846	12,598	12,398	12,158	11,005	7,734	68,739	
**Total_Human**	H3N2	17,179	16,856	16,856	28,158	14,385	20,386	113,820	217,549
**Total_Others**	Others	4,379	4,421	4,168	9,113	4,427	8,482	34,990	
**Total**	/	34,404	33,875	33,422	49,429	29,817	36,602	217,549	/

Supplemental Table 2 Cross_validation score and its moving average level for PB2 genomic sequences by Gradient Boosted Regression Trees (GBRT), Multiple Layer Perception Classifier (MLP), Random Forest Classifier (RFC) and support vector classifier (SVC).

	GBRT		MLP		RFC		SVC	
(d)nt_num	cv_score	MA3_cv_score	cv_score	MA3_cv_score	cv_score	MA3_cv_score	cv_score	MA3_cv_score
0	0.872	0.872	0.839	0.839	0.872	0.872	0.571	0.571
1	0.964	0.918	0.941	0.89	0.963	0.918	0.891	0.731
2	0.979	0.938	0.961	0.914	0.98	0.939	0.865	0.776
3	0.992	0.978	0.983	0.962	0.993	0.979	0.913	0.89
4	0.993	0.988	0.977	0.974	0.993	0.989	0.934	0.904
5	0.994	0.993	0.979	0.98	0.995	0.994	0.971	0.939
6	0.995	0.994	0.978	0.978	0.995	0.994	0.966	0.957
7	0.995	0.994	0.984	0.98	0.995	0.995	0.966	0.968
8	0.995	0.995	0.967	0.977	0.995	0.995	0.967	0.966
9	0.995	0.995	0.986	0.979	0.996	0.995	0.969	0.967
10	0.995	0.995	0.988	0.98	0.996	0.995	0.972	0.969
11	0.995	0.995	0.989	0.988	0.995	0.995	0.976	0.972
12	0.995	0.995	0.991	0.989	0.996	0.995	0.972	0.973
13	0.995	0.995	0.987	0.989	0.996	0.995	0.974	0.974
14	0.995	0.995	0.99	0.989	0.995	0.995	0.974	0.973
15	0.995	0.995	0.987	0.988	0.995	0.995	0.972	0.973
16	0.995	0.995	0.99	0.989	0.995	0.995	0.989	0.978
17	0.995	0.995	0.99	0.989	0.995	0.995	0.989	0.983
18	0.995	0.995	0.988	0.99	0.995	0.995	0.989	0.989
19	0.994	0.994	0.988	0.989	0.996	0.995	0.989	0.989
20	0.995	0.994	0.989	0.989	0.996	0.996	0.99	0.989
21	0.995	0.994	0.989	0.989	0.996	0.996	0.99	0.99
22	0.995	0.995	0.99	0.99	0.996	0.996	0.99	0.99
23	0.995	0.995	0.99	0.99	0.995	0.996	0.99	0.99
24	0.995	0.995	0.99	0.99	0.996	0.996	0.99	0.99
25	0.995	0.995	0.992	0.99	0.995	0.996	0.99	0.99
26	0.995	0.995	0.989	0.99	0.995	0.996	0.99	0.99
27	0.995	0.995	0.989	0.99	0.996	0.996	0.99	0.99
28	0.995	0.995	0.989	0.989	0.995	0.995	0.99	0.99
29	0.995	0.995	0.988	0.989	0.996	0.996	0.99	0.99
30	0.995	0.995	0.99	0.989	0.996	0.996	0.989	0.99
31	0.995	0.995	0.99	0.99	0.996	0.996	0.99	0.99
32	0.995	0.995	0.989	0.99	0.996	0.996	0.99	0.99
33	0.994	0.995	0.99	0.99	0.996	0.996	0.991	0.99
34	0.995	0.995	0.99	0.989	0.995	0.996	0.99	0.99
35	0.995	0.995	0.99	0.99	0.996	0.996	0.99	0.99
36	0.995	0.995	0.993	0.991	0.996	0.996	0.991	0.991

37	0.995	0.995	0.992	0.992	0.996	0.996	0.991	0.991
38	0.995	0.995	0.99	0.992	0.996	0.996	0.991	0.991
39	0.995	0.995	0.993	0.992	0.996	0.996	0.991	0.991
40	0.996	0.995	0.991	0.991	0.996	0.996	0.991	0.991
41	0.996	0.996	0.993	0.992	0.995	0.996	0.991	0.991
42	0.994	0.995	0.987	0.99	0.996	0.996	0.991	0.991
43	0.996	0.995	0.991	0.99	0.996	0.996	0.991	0.991
44	0.995	0.995	0.99	0.989	0.996	0.996	0.991	0.991
45	0.995	0.995	0.99	0.99	0.996	0.996	0.991	0.991
46	0.996	0.995	0.994	0.991	0.996	0.996	0.991	0.991
47	0.995	0.995	0.991	0.991	0.996	0.996	0.991	0.991
48	0.995	0.995	0.992	0.992	0.996	0.996	0.991	0.991
49	0.996	0.995	0.994	0.992	0.995	0.996	0.991	0.991
50	0.995	0.995	0.992	0.993	0.996	0.996	0.991	0.991
51	0.995	0.995	0.991	0.992	0.996	0.996	0.991	0.991
52	0.995	0.995	0.993	0.992	0.996	0.996	0.991	0.991
53	0.994	0.995	0.989	0.991	0.996	0.996	0.992	0.991
54	0.995	0.995	0.992	0.991	0.996	0.996	0.993	0.992
55	0.992	0.994	0.992	0.991	0.996	0.996	0.993	0.992
56	0.992	0.993	0.989	0.991	0.995	0.996	0.993	0.993
57	0.992	0.992	0.994	0.992	0.996	0.996	0.993	0.993
58	0.994	0.993	0.991	0.991	0.996	0.996	0.993	0.993
59	0.992	0.993	0.992	0.992	0.996	0.996	0.993	0.993

Supplemental Table 3 Cross_validation score and its moving average level for PB1 genomic sequences by GBRT, MLP, RFC and SVC.

	GBRT		MLP		RFC		SVC	
(d)nt_num	cv_score	MA3_cv_score	cv_score	MA3_cv_score	cv_score	MA3_cv_score	cv_score	MA3_cv_score
0	0.912	0.912	0.88	0.88	0.92	0.92	0.878	0.878
1	0.97	0.941	0.963	0.922	0.971	0.946	0.931	0.904
2	0.981	0.954	0.973	0.939	0.983	0.958	0.934	0.914
3	0.984	0.978	0.972	0.969	0.985	0.98	0.935	0.933
4	0.984	0.983	0.976	0.974	0.988	0.985	0.939	0.936
5	0.985	0.984	0.976	0.975	0.989	0.987	0.939	0.938
6	0.985	0.985	0.971	0.974	0.989	0.988	0.962	0.947
7	0.987	0.985	0.973	0.973	0.99	0.989	0.965	0.955
8	0.989	0.987	0.98	0.974	0.992	0.99	0.975	0.967
9	0.994	0.99	0.987	0.98	0.993	0.992	0.984	0.975
10	0.994	0.992	0.98	0.982	0.995	0.993	0.982	0.98
11	0.995	0.994	0.989	0.985	0.994	0.994	0.987	0.984
12	0.994	0.995	0.988	0.986	0.994	0.995	0.987	0.985
13	0.995	0.995	0.99	0.989	0.994	0.994	0.988	0.987
14	0.995	0.995	0.99	0.989	0.995	0.994	0.988	0.988

15	0.995	0.995	0.99	0.99	0.995	0.994	0.988	0.988
16	0.995	0.995	0.991	0.99	0.995	0.995	0.988	0.988
17	0.995	0.995	0.99	0.99	0.995	0.995	0.988	0.988
18	0.996	0.995	0.99	0.99	0.995	0.995	0.987	0.988
19	0.996	0.995	0.99	0.99	0.995	0.995	0.988	0.988
20	0.995	0.995	0.989	0.99	0.995	0.995	0.988	0.988
21	0.995	0.995	0.99	0.99	0.994	0.995	0.989	0.988
22	0.995	0.995	0.992	0.99	0.995	0.995	0.989	0.989
23	0.996	0.995	0.992	0.991	0.994	0.995	0.988	0.989
24	0.995	0.995	0.994	0.993	0.995	0.995	0.988	0.988
25	0.996	0.996	0.996	0.994	0.995	0.995	0.988	0.988
26	0.995	0.996	0.993	0.994	0.995	0.995	0.988	0.988
27	0.996	0.996	0.995	0.995	0.994	0.995	0.988	0.988
28	0.996	0.996	0.995	0.994	0.994	0.995	0.99	0.989
29	0.996	0.996	0.993	0.995	0.996	0.995	0.99	0.989
30	0.996	0.996	0.994	0.994	0.995	0.995	0.99	0.99
31	0.996	0.996	0.995	0.994	0.996	0.995	0.99	0.99
32	0.996	0.996	0.995	0.995	0.995	0.995	0.99	0.99
33	0.996	0.996	0.996	0.995	0.996	0.995	0.99	0.99
34	0.996	0.996	0.992	0.994	0.995	0.995	0.99	0.99
35	0.996	0.996	0.996	0.995	0.995	0.995	0.99	0.99
36	0.996	0.996	0.993	0.994	0.995	0.995	0.99	0.99
37	0.996	0.996	0.994	0.994	0.996	0.995	0.99	0.99
38	0.996	0.996	0.996	0.994	0.996	0.995	0.99	0.99
39	0.996	0.996	0.99	0.993	0.995	0.995	0.99	0.99
40	0.996	0.996	0.993	0.993	0.995	0.995	0.99	0.99
41	0.996	0.996	0.994	0.992	0.995	0.995	0.99	0.99
42	0.996	0.996	0.994	0.994	0.995	0.995	0.991	0.99
43	0.996	0.996	0.995	0.995	0.996	0.995	0.991	0.99
44	0.997	0.996	0.993	0.994	0.996	0.996	0.99	0.991
45	0.997	0.996	0.995	0.995	0.996	0.996	0.99	0.991
46	0.996	0.997	0.995	0.994	0.996	0.996	0.991	0.991
47	0.996	0.996	0.996	0.995	0.995	0.996	0.991	0.991
48	0.995	0.996	0.993	0.994	0.996	0.996	0.991	0.991
49	0.995	0.996	0.996	0.995	0.996	0.996	0.991	0.991
50	0.995	0.995	0.993	0.994	0.995	0.996	0.991	0.991
51	0.996	0.996	0.994	0.994	0.996	0.996	0.991	0.991
52	0.996	0.996	0.995	0.994	0.996	0.996	0.991	0.991
53	0.996	0.996	0.992	0.994	0.995	0.996	0.991	0.991
54	0.996	0.996	0.995	0.994	0.996	0.996	0.992	0.991
55	0.996	0.996	0.993	0.993	0.996	0.996	0.992	0.992
56	0.995	0.996	0.996	0.994	0.996	0.996	0.992	0.992
57	0.996	0.996	0.992	0.994	0.996	0.996	0.992	0.992
58	0.995	0.995	0.993	0.994	0.996	0.996	0.992	0.992

59	0.996	0.996	0.996	0.994	0.996	0.996	0.992	0.992

* PB1, mlp,cv_score,rolling, amended value = 10

Supplemental Table 4 Cross_validation score and its moving average level for PA genomic sequences by GBRT, MLP, RFC and SVC.

	GBRT		MLP		RFC		SVC	
(d)nt_num	cv_score	MA3_cv_score	cv_score	MA3_cv_score	cv_score	MA3_cv_score	cv_score	MA3_cv_score
0	0.972	0.972	0.972	0.972	0.972	0.972	0.573	0.573
1	0.974	0.973	0.978	0.975	0.975	0.973	0.573	0.573
2	0.989	0.979	0.957	0.969	0.987	0.978	0.787	0.644
3	0.99	0.984	0.937	0.957	0.989	0.984	0.854	0.738
4	0.989	0.989	0.963	0.952	0.989	0.989	0.855	0.832
5	0.987	0.989	0.94	0.947	0.99	0.989	0.843	0.851
6	0.987	0.988	0.939	0.947	0.99	0.99	0.865	0.854
7	0.988	0.987	0.96	0.946	0.991	0.99	0.933	0.88
8	0.987	0.988	0.963	0.954	0.99	0.99	0.934	0.91
9	0.99	0.989	0.966	0.963	0.992	0.991	0.929	0.932
10	0.988	0.989	0.966	0.965	0.991	0.991	0.95	0.938
11	0.988	0.989	0.963	0.965	0.991	0.991	0.942	0.94
12	0.989	0.989	0.974	0.968	0.992	0.991	0.941	0.945
13	0.991	0.989	0.983	0.973	0.992	0.992	0.96	0.948
14	0.993	0.991	0.984	0.98	0.993	0.993	0.96	0.954
15	0.992	0.992	0.978	0.981	0.992	0.993	0.96	0.96
16	0.993	0.992	0.978	0.98	0.993	0.993	0.96	0.96
17	0.993	0.993	0.981	0.979	0.993	0.993	0.967	0.962
18	0.992	0.993	0.988	0.982	0.993	0.993	0.971	0.966
19	0.993	0.993	0.985	0.984	0.993	0.993	0.972	0.97
20	0.993	0.993	0.987	0.987	0.993	0.993	0.978	0.974
21	0.994	0.993	0.983	0.985	0.994	0.993	0.978	0.976
22	0.994	0.994	0.986	0.985	0.993	0.993	0.98	0.979
23	0.994	0.994	0.988	0.986	0.994	0.994	0.981	0.98
24	0.993	0.994	0.985	0.987	0.993	0.994	0.98	0.98
25	0.992	0.993	0.988	0.987	0.994	0.994	0.978	0.98
26	0.994	0.993	0.978	0.984	0.994	0.994	0.976	0.978
27	0.993	0.993	0.984	0.984	0.994	0.994	0.977	0.977
28	0.992	0.993	0.986	0.983	0.994	0.994	0.977	0.977
29	0.993	0.993	0.983	0.984	0.994	0.994	0.979	0.977
30	0.992	0.992	0.995	0.988	0.994	0.994	0.987	0.981
31	0.992	0.992	0.991	0.99	0.994	0.994	0.987	0.984
32	0.992	0.992	0.993	0.993	0.994	0.994	0.987	0.987
33	0.993	0.992	0.994	0.993	0.994	0.994	0.989	0.988
34	0.992	0.992	0.994	0.994	0.993	0.994	0.989	0.988
35	0.992	0.993	0.988	0.992	0.994	0.994	0.99	0.989

36	0.992	0.992	0.995	0.992	0.994	0.994	0.99	0.989
37	0.992	0.992	0.989	0.991	0.994	0.994	0.991	0.99
38	0.992	0.992	0.994	0.993	0.994	0.994	0.991	0.991
39	0.992	0.992	0.992	0.991	0.994	0.994	0.991	0.991
40	0.992	0.992	0.994	0.993	0.994	0.994	0.991	0.991
41	0.992	0.992	0.994	0.993	0.994	0.994	0.991	0.991
42	0.992	0.992	0.994	0.994	0.994	0.994	0.993	0.992
43	0.991	0.992	0.991	0.993	0.994	0.994	0.993	0.992
44	0.991	0.991	0.995	0.993	0.994	0.994	0.993	0.993
45	0.992	0.992	0.994	0.993	0.994	0.994	0.992	0.993
46	0.991	0.992	0.993	0.994	0.994	0.994	0.993	0.993
47	0.992	0.992	0.994	0.994	0.995	0.995	0.993	0.993
48	0.991	0.991	0.994	0.994	0.994	0.994	0.993	0.993
49	0.99	0.991	0.994	0.994	0.994	0.994	0.993	0.993
50	0.99	0.991	0.993	0.994	0.994	0.994	0.993	0.993
51	0.991	0.991	0.995	0.994	0.994	0.994	0.993	0.993
52	0.992	0.991	0.995	0.994	0.994	0.994	0.993	0.993
53	0.992	0.992	0.993	0.994	0.994	0.994	0.993	0.993
54	0.992	0.992	0.992	0.993	0.994	0.994	0.993	0.993
55	0.991	0.992	0.995	0.993	0.994	0.994	0.993	0.993
56	0.991	0.991	0.995	0.994	0.994	0.994	0.993	0.993
57	0.992	0.991	0.993	0.994	0.994	0.994	0.993	0.993
58	0.992	0.991	0.994	0.994	0.994	0.994	0.994	0.993
59	0.992	0.992	0.996	0.994	0.995	0.994	0.994	0.994

Supplemental Table 5 Cross_validation score and its moving average level for HA genomic sequences by GBRT, MLP, RFC and SVC.

	GBRT		MLP		RFC		SVC	
(d)nt_num	cv_score	MA3_cv_score	cv_score	MA3_cv_score	cv_score	MA3_cv_score	cv_score	MA3_cv_score
0	0.785	0.785	0.647	0.647	0.895	0.895	0.696	0.696
1	0.881	0.833	0.827	0.737	0.935	0.915	0.800	0.748
2	0.960	0.875	0.871	0.781	0.976	0.936	0.814	0.770
3	0.965	0.936	0.888	0.862	0.980	0.964	0.840	0.818
4	0.979	0.968	0.931	0.897	0.987	0.981	0.835	0.829
5	0.987	0.977	0.953	0.924	0.993	0.987	0.898	0.858
6	0.991	0.986	0.962	0.949	0.994	0.991	0.906	0.880
7	0.993	0.990	0.962	0.959	0.995	0.994	0.917	0.907
8	0.994	0.993	0.964	0.963	0.995	0.995	0.916	0.913
9	0.994	0.994	0.979	0.968	0.995	0.995	0.918	0.917
10	0.995	0.994	0.980	0.974	0.995	0.995	0.918	0.917
11	0.995	0.995	0.982	0.980	0.996	0.995	0.943	0.926
12	0.993	0.994	0.985	0.982	0.996	0.995	0.961	0.941
13	0.993	0.994	0.986	0.984	0.997	0.996	0.968	0.957

14	0.993	0.993	0.985	0.985	0.996	0.996	0.968	0.966
15	0.992	0.993	0.989	0.987	0.996	0.996	0.970	0.969
16	0.995	0.993	0.992	0.989	0.996	0.996	0.976	0.971
17	0.995	0.994	0.993	0.992	0.996	0.996	0.978	0.975
18	0.995	0.995	0.989	0.991	0.996	0.996	0.978	0.978
19	0.994	0.995	0.992	0.991	0.997	0.996	0.979	0.978
20	0.995	0.995	0.992	0.991	0.997	0.997	0.981	0.979
21	0.995	0.995	0.990	0.991	0.997	0.997	0.982	0.981
22	0.995	0.995	0.993	0.992	0.997	0.997	0.981	0.982
23	0.995	0.995	0.993	0.992	0.997	0.997	0.982	0.982
24	0.995	0.995	0.994	0.994	0.997	0.997	0.984	0.983
25	0.992	0.994	0.994	0.994	0.997	0.997	0.984	0.983
26	0.996	0.994	0.993	0.994	0.997	0.997	0.984	0.984
27	0.995	0.994	0.993	0.993	0.997	0.997	0.986	0.985
28	0.995	0.995	0.993	0.993	0.997	0.997	0.986	0.985
29	0.995	0.995	0.988	0.991	0.997	0.997	0.986	0.986
30	0.996	0.995	0.993	0.991	0.997	0.997	0.988	0.987
31	0.996	0.996	0.993	0.991	0.997	0.997	0.988	0.987
32	0.996	0.996	0.994	0.993	0.997	0.997	0.989	0.988
33	0.996	0.996	0.994	0.994	0.997	0.997	0.989	0.989
34	0.996	0.996	0.990	0.993	0.996	0.997	0.989	0.989
35	0.996	0.996	0.994	0.993	0.997	0.997	0.990	0.990
36	0.997	0.996	0.993	0.992	0.997	0.997	0.992	0.990
37	0.996	0.996	0.995	0.994	0.997	0.997	0.992	0.991
38	0.997	0.997	0.996	0.995	0.997	0.997	0.993	0.992
39	0.997	0.997	0.996	0.996	0.997	0.997	0.993	0.992
40	0.997	0.997	0.994	0.995	0.997	0.997	0.993	0.993
41	0.997	0.997	0.994	0.995	0.997	0.997	0.993	0.993
42	0.997	0.997	0.995	0.994	0.996	0.997	0.993	0.993
43	0.997	0.997	0.995	0.994	0.997	0.997	0.994	0.993
44	0.997	0.997	0.994	0.995	0.997	0.997	0.993	0.993
45	0.997	0.997	0.995	0.994	0.997	0.997	0.993	0.993
46	0.996	0.997	0.994	0.994	0.997	0.997	0.993	0.993
47	0.996	0.996	0.996	0.995	0.997	0.997	0.993	0.993
48	0.997	0.996	0.995	0.995	0.997	0.997	0.993	0.993
49	0.997	0.997	0.996	0.996	0.997	0.997	0.993	0.993
50	0.996	0.997	0.994	0.995	0.997	0.997	0.993	0.993
51	0.997	0.997	0.996	0.995	0.997	0.997	0.993	0.993
52	0.996	0.996	0.994	0.995	0.997	0.997	0.993	0.993
53	0.996	0.996	0.995	0.995	0.997	0.997	0.993	0.993
54	0.997	0.996	0.995	0.995	0.997	0.997	0.994	0.994
55	0.997	0.997	0.996	0.995	0.997	0.997	0.994	0.994
56	0.997	0.997	0.996	0.996	0.997	0.997	0.994	0.994
57	0.997	0.997	0.995	0.996	0.997	0.997	0.994	0.994

58	0.996	0.996	0.995	0.995	0.997	0.997	0.994	0.994
59	0.997	0.996	0.996	0.995	0.997	0.997	0.994	0.994

Supplemental Table 6 Cross_validation score and its moving average level for NP genomic sequences by GBRT, MLP, RFC and SVC.

	GBRT		MLP		RFC		SVC	
(d)nt_num	cv_score	MA3_cv_score	cv_score	MA3_cv_score	cv_score	MA3_cv_score	cv_score	MA3_cv_score
0	0.737	0.737	0.668	0.668	0.822	0.822	0.657	0.657
1	0.916	0.827	0.829	0.749	0.952	0.887	0.664	0.661
2	0.938	0.864	0.794	0.764	0.966	0.913	0.673	0.665
3	0.974	0.942	0.921	0.848	0.977	0.965	0.616	0.651
4	0.991	0.968	0.967	0.894	0.993	0.979	0.859	0.716
5	0.994	0.986	0.972	0.954	0.995	0.988	0.962	0.812
6	0.995	0.993	0.982	0.974	0.995	0.994	0.982	0.934
7	0.995	0.995	0.993	0.982	0.995	0.995	0.994	0.979
8	0.994	0.994	0.993	0.989	0.995	0.995	0.994	0.99
9	0.993	0.994	0.994	0.993	0.995	0.995	0.995	0.994
10	0.993	0.993	0.994	0.994	0.995	0.995	0.995	0.995
11	0.994	0.993	0.994	0.994	0.995	0.995	0.995	0.995
12	0.994	0.994	0.994	0.994	0.995	0.995	0.995	0.995
13	0.994	0.994	0.994	0.994	0.995	0.995	0.995	0.995
14	0.995	0.995	0.994	0.994	0.996	0.995	0.995	0.995
15	0.995	0.995	0.994	0.994	0.996	0.996	0.995	0.995
16	0.994	0.995	0.994	0.994	0.995	0.995	0.995	0.995
17	0.994	0.994	0.993	0.994	0.996	0.995	0.995	0.995
18	0.994	0.994	0.994	0.994	0.996	0.996	0.995	0.995
19	0.994	0.994	0.994	0.994	0.996	0.996	0.996	0.995
20	0.994	0.994	0.994	0.994	0.996	0.996	0.996	0.995
21	0.994	0.994	0.993	0.994	0.996	0.996	0.996	0.996
22	0.994	0.994	0.993	0.993	0.996	0.996	0.996	0.996
23	0.993	0.994	0.992	0.993	0.996	0.996	0.996	0.996
24	0.994	0.994	0.994	0.993	0.996	0.996	0.996	0.996
25	0.994	0.994	0.994	0.994	0.996	0.996	0.995	0.996
26	0.994	0.994	0.993	0.994	0.995	0.996	0.996	0.996
27	0.994	0.994	0.993	0.994	0.996	0.996	0.996	0.996
28	0.994	0.994	0.994	0.994	0.995	0.995	0.996	0.996
29	0.993	0.994	0.995	0.994	0.996	0.996	0.996	0.996
30	0.994	0.994	0.994	0.994	0.995	0.995	0.996	0.996
31	0.993	0.994	0.995	0.994	0.996	0.996	0.996	0.996
32	0.993	0.994	0.994	0.994	0.995	0.995	0.996	0.996
33	0.994	0.994	0.994	0.994	0.995	0.995	0.996	0.996
34	0.994	0.994	0.995	0.994	0.995	0.995	0.995	0.996
35	0.993	0.994	0.994	0.994	0.996	0.995	0.995	0.995

36	0.994	0.994	0.995	0.994	0.996	0.995	0.996	0.996
37	0.993	0.994	0.994	0.994	0.996	0.996	0.996	0.996
38	0.993	0.994	0.995	0.995	0.995	0.996	0.996	0.996
39	0.994	0.993	0.994	0.994	0.996	0.996	0.996	0.996
40	0.994	0.994	0.995	0.994	0.995	0.995	0.996	0.996
41	0.994	0.994	0.995	0.994	0.995	0.995	0.996	0.996
42	0.994	0.994	0.994	0.994	0.995	0.995	0.996	0.996
43	0.993	0.993	0.995	0.994	0.996	0.995	0.996	0.996
44	0.993	0.993	0.994	0.994	0.995	0.995	0.996	0.996
45	0.994	0.993	0.994	0.994	0.996	0.996	0.996	0.996
46	0.992	0.993	0.994	0.994	0.996	0.995	0.996	0.996
47	0.994	0.993	0.993	0.994	0.995	0.995	0.996	0.996
48	0.994	0.993	0.994	0.994	0.995	0.995	0.996	0.996
49	0.994	0.994	0.994	0.994	0.995	0.995	0.996	0.996
50	0.994	0.994	0.994	0.994	0.996	0.995	0.996	0.996
51	0.994	0.994	0.995	0.994	0.995	0.995	0.996	0.996
52	0.993	0.994	0.993	0.994	0.994	0.995	0.996	0.996
53	0.995	0.994	0.993	0.994	0.995	0.995	0.996	0.996
54	0.994	0.994	0.995	0.994	0.995	0.995	0.996	0.996
55	0.995	0.995	0.995	0.994	0.996	0.995	0.996	0.996
56	0.994	0.994	0.996	0.995	0.996	0.995	0.996	0.996
57	0.995	0.995	0.994	0.995	0.995	0.995	0.996	0.996
58	0.994	0.994	0.994	0.995	0.995	0.995	0.996	0.996
59	0.995	0.995	0.995	0.994	0.995	0.995	0.996	0.996

Supplemental Table 7 Cross_validation score and its moving average level for NA genomic sequences by GBRT, MLP, RFC and SVC.

	GBRT		MLP		RFC		SVC	
(d)nt_num	cv_score	MA3_cv_score	cv_score	MA3_cv_score	cv_score	MA3_cv_score	cv_score	MA3_cv_score
0	0.93	0.93	0.909	0.909	0.943	0.943	0.905	0.905
1	0.954	0.942	0.939	0.924	0.962	0.953	0.912	0.908
2	0.984	0.956	0.976	0.941	0.986	0.964	0.973	0.93
3	0.987	0.975	0.978	0.964	0.987	0.979	0.973	0.953
4	0.987	0.986	0.978	0.978	0.988	0.987	0.978	0.975
5	0.992	0.989	0.988	0.981	0.993	0.99	0.988	0.98
6	0.993	0.991	0.989	0.985	0.994	0.992	0.989	0.985
7	0.993	0.993	0.989	0.989	0.994	0.994	0.989	0.988
8	0.993	0.993	0.989	0.989	0.994	0.994	0.989	0.989
9	0.993	0.993	0.988	0.989	0.994	0.994	0.988	0.989
10	0.993	0.993	0.987	0.988	0.994	0.994	0.989	0.989
11	0.994	0.993	0.988	0.988	0.995	0.994	0.989	0.989
12	0.994	0.994	0.991	0.989	0.995	0.995	0.991	0.99
13	0.994	0.994	0.99	0.99	0.994	0.995	0.991	0.99

14	0.994	0.994	0.991	0.991	0.995	0.995	0.991	0.991
15	0.995	0.994	0.992	0.991	0.995	0.995	0.994	0.992
16	0.994	0.994	0.993	0.992	0.995	0.995	0.994	0.993
17	0.994	0.994	0.993	0.993	0.995	0.995	0.994	0.994
18	0.994	0.994	0.99	0.992	0.995	0.995	0.994	0.994
19	0.994	0.994	0.991	0.991	0.995	0.995	0.994	0.994
20	0.994	0.994	0.992	0.991	0.995	0.995	0.994	0.994
21	0.994	0.994	0.994	0.992	0.995	0.995	0.994	0.994
22	0.994	0.994	0.992	0.992	0.995	0.995	0.995	0.994
23	0.995	0.994	0.994	0.993	0.996	0.995	0.995	0.995
24	0.994	0.994	0.993	0.993	0.995	0.995	0.995	0.995
25	0.994	0.994	0.994	0.994	0.995	0.996	0.995	0.995
26	0.994	0.994	0.994	0.994	0.996	0.996	0.995	0.995
27	0.993	0.994	0.993	0.994	0.996	0.996	0.995	0.995
28	0.994	0.994	0.994	0.994	0.996	0.996	0.995	0.995
29	0.994	0.994	0.994	0.994	0.995	0.996	0.995	0.995
30	0.994	0.994	0.995	0.994	0.995	0.995	0.995	0.995
31	0.994	0.994	0.992	0.993	0.995	0.995	0.995	0.995
32	0.995	0.994	0.995	0.994	0.996	0.995	0.995	0.995
33	0.994	0.994	0.994	0.994	0.995	0.995	0.995	0.995
34	0.994	0.994	0.994	0.994	0.996	0.995	0.995	0.995
35	0.994	0.994	0.995	0.995	0.995	0.995	0.995	0.995
36	0.993	0.994	0.994	0.994	0.996	0.996	0.995	0.995
37	0.995	0.994	0.994	0.994	0.996	0.996	0.995	0.995
38	0.994	0.994	0.993	0.994	0.995	0.996	0.995	0.995
39	0.994	0.994	0.992	0.993	0.995	0.996	0.995	0.995
40	0.994	0.994	0.993	0.993	0.995	0.995	0.995	0.995
41	0.993	0.994	0.993	0.992	0.995	0.995	0.995	0.995
42	0.994	0.993	0.994	0.993	0.996	0.995	0.995	0.995
43	0.994	0.994	0.993	0.993	0.996	0.996	0.995	0.995
44	0.995	0.994	0.991	0.993	0.996	0.996	0.995	0.995
45	0.994	0.994	0.994	0.993	0.996	0.996	0.995	0.995
46	0.994	0.994	0.994	0.993	0.995	0.995	0.995	0.995
47	0.995	0.994	0.993	0.994	0.996	0.996	0.995	0.995
48	0.995	0.995	0.994	0.994	0.997	0.996	0.995	0.995
49	0.995	0.995	0.993	0.993	0.996	0.996	0.995	0.995
50	0.995	0.995	0.994	0.993	0.995	0.996	0.995	0.995
51	0.995	0.995	0.991	0.993	0.996	0.995	0.995	0.995
52	0.995	0.995	0.993	0.993	0.995	0.995	0.995	0.995
53	0.995	0.995	0.993	0.992	0.995	0.996	0.995	0.995
54	0.995	0.995	0.994	0.993	0.996	0.996	0.995	0.995
55	0.995	0.995	0.99	0.992	0.996	0.996	0.995	0.995
56	0.994	0.995	0.991	0.992	0.997	0.996	0.995	0.995
57	0.995	0.995	0.991	0.991	0.996	0.996	0.995	0.995

58	0.995	0.995	0.993	0.992	0.996	0.996	0.995	0.995
59	0.995	0.995	0.992	0.992	0.996	0.996	0.995	0.995

Supplemental Methods

Supplemental information about Feature Engineering and sequence resampling

Supplemental information about Feature Engineering

Feature engineering and feature selection were most important for machine learning analysis. Biologically, there is a species barrier for human and avian influenza viruses, and there should be a linear separability of genotype and phenotype between both groups of viruses. Here, we supposed that the genomic composition of mono- or di-nucleotide is associated with the linear separability. In another word, there should be a hyper plane with a margin between avian and human viruses in genomic composition. We supposed that the human/avian-IAV-separability should be consistently linear and make sense biologically. In this context, support vector classifier (SVC) was the best choice. In the case of SVC, data points are viewed as n-dimensional vectors multiply m-number, and it is to separate such points with a hyperplane with maximum-margin. The nonlinear separators, Gradient Boosted Regression Trees (GBRT), Random Forest Classifier (RFC) and Multiple Layer Perception Classifier (MLP), which are based on neural network (MLP) or decision tree (RFC and GBRT), are grown very deep tend to learn highly irregular patterns, at the expense of a small increase in the bias and some loss of interpretability, let alone the biological separability. However, to avoid over-fitting, we adjusted the optimized (d)nt number of SVC, via averaging it with the optimized (d)nt number with MLP, RFC and GBRT classifiers.

SVC was the optional model. Thus, SVC was used as main supervised machine learning model for both feature selection and sample classification. SVC was used firstly for (d)nt sorting, secondly for (d)nt optimization, along with principal component analysis (PCA), thirdly as train final classifier with the optimized (d)nts. The (d)nt optimization was performed using four types of machine learning approaches, SVC, GBRT, RFC and MLP. methods.

As Supplementary Figure 3 shown, avian and human sequences were not well classified separately with the 60 (d)nt features. Moreover, as compositional information, the 60 (d)nt features were theoretically not independent of each other, and there was a feature redundancy for the 60 (d)nts. Thus, PCA is used to reduce the dimensionality of batches of (d)nt features before SVC analysis for the feature selection. If there was a higher dependence/correlation between/among a batch of (d)nt features, the AUC score of SVC would be lower post dimensionality reduction of (d)nt features by PCA. In addition, it is time-saving for the calculation of only one PCA value, rather a feature matrix.

Theoretically, to identify every possible dependence of (d)nt features, every possible combination of (d)nt features, with various feature number (a combination of m features from n features, 2< = m <= 30,since combination (60,m)= combination (60,(60-m))), should be utilized for the PCA/SVC feature selection. However, it is a huge job to exhaust all combinations. Here, we selected 2*combination (60, 2) (3,540) as sampling times for a random sampling of four features from the 60 features for the feature

selection with PCA/SVC. As shown in Supplementary Figure 6, more than 200 times were sampled for each of the 60 features in such process. For each time of PCA/SVC analysis, AUC score was taken as the feature importance value for each of the four sampled features. According to the average (n>200) AUC score, the 60 (d)nt features were sorted.

Finally, SVC, MLP, RFC and GBRT with accumulating (d)nt features were performed again for (d)nt number optimization. The feature list was updated for each round of SVC analysis, with top n (n = n +1 for n in range [1,59]) (d)nt features from the sorted feature list. The 60 AUC score value of the 60 iteration of machine learning analysis were utilized for the final (d)nt number optimization.

Supplemental information about sequence resampling

Resampling was performed via pandas.DataFrame.sample (Python) with a float ratio multiplying the segment sequence number, and the final sequence number was an integral number (the Integral function in python is just removing the float, not same as the Rounding function). Thus, 59-61 segment sequence samples were produced for phylogeny and hierarchical clustering analysis, 46, 042 human-adaptive sequences and 46, 488 human-inadaptive avian sequences were produced for feature extraction and model building, with not the same sample number for avian and human sets.

Supporting data

Supporting data includes the sequence ID table, the supporting data for Figures and for supplementary Figures. Supporting data was available online: https://github.com/Jamalijama/Predict_IAV_Host.

Code availability

The project code available at following website:

https://github.com/Jamalijama/Predict_IAV_Host.

Supplemental Figure 1

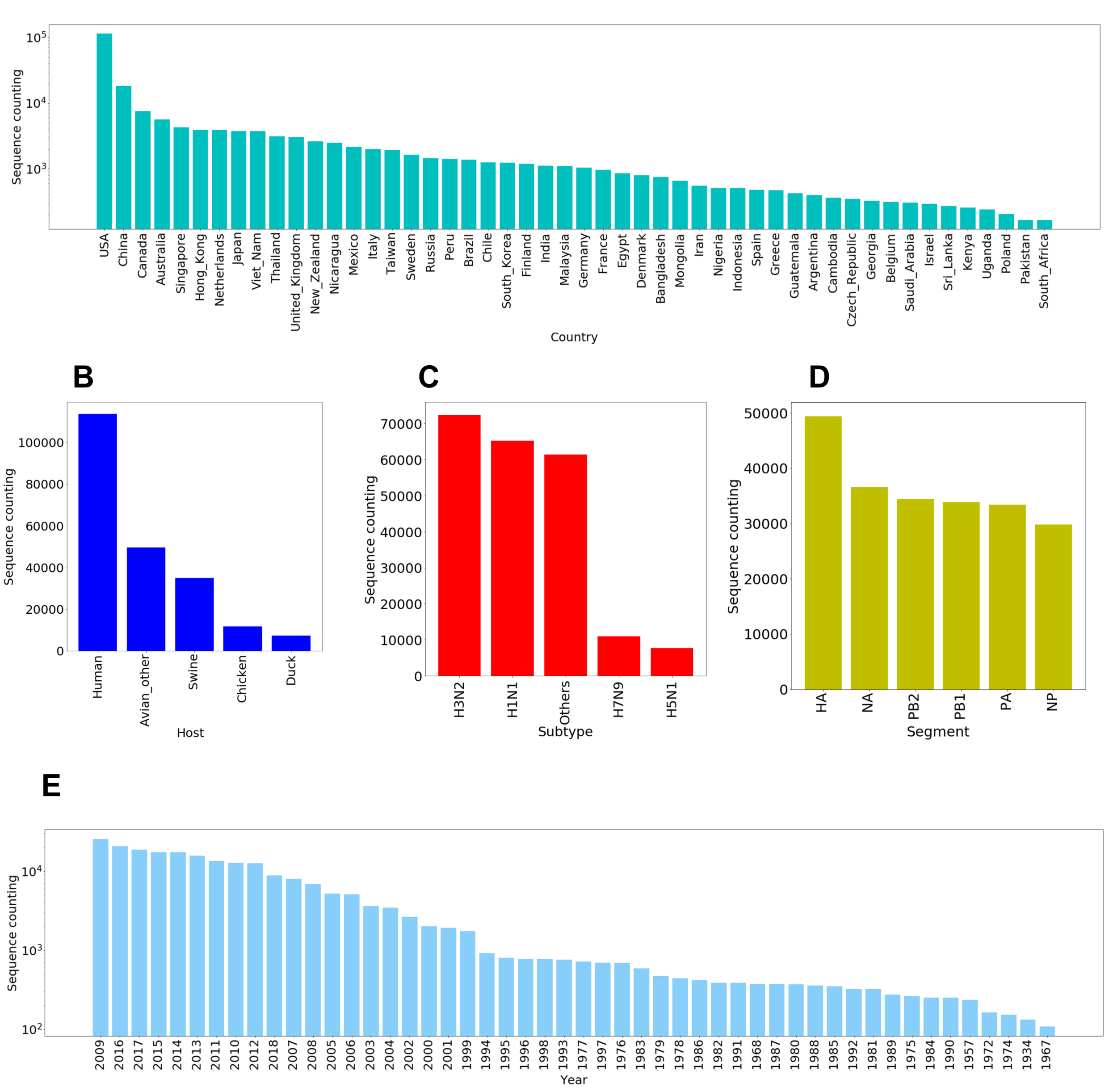

Supplemental Figure 2

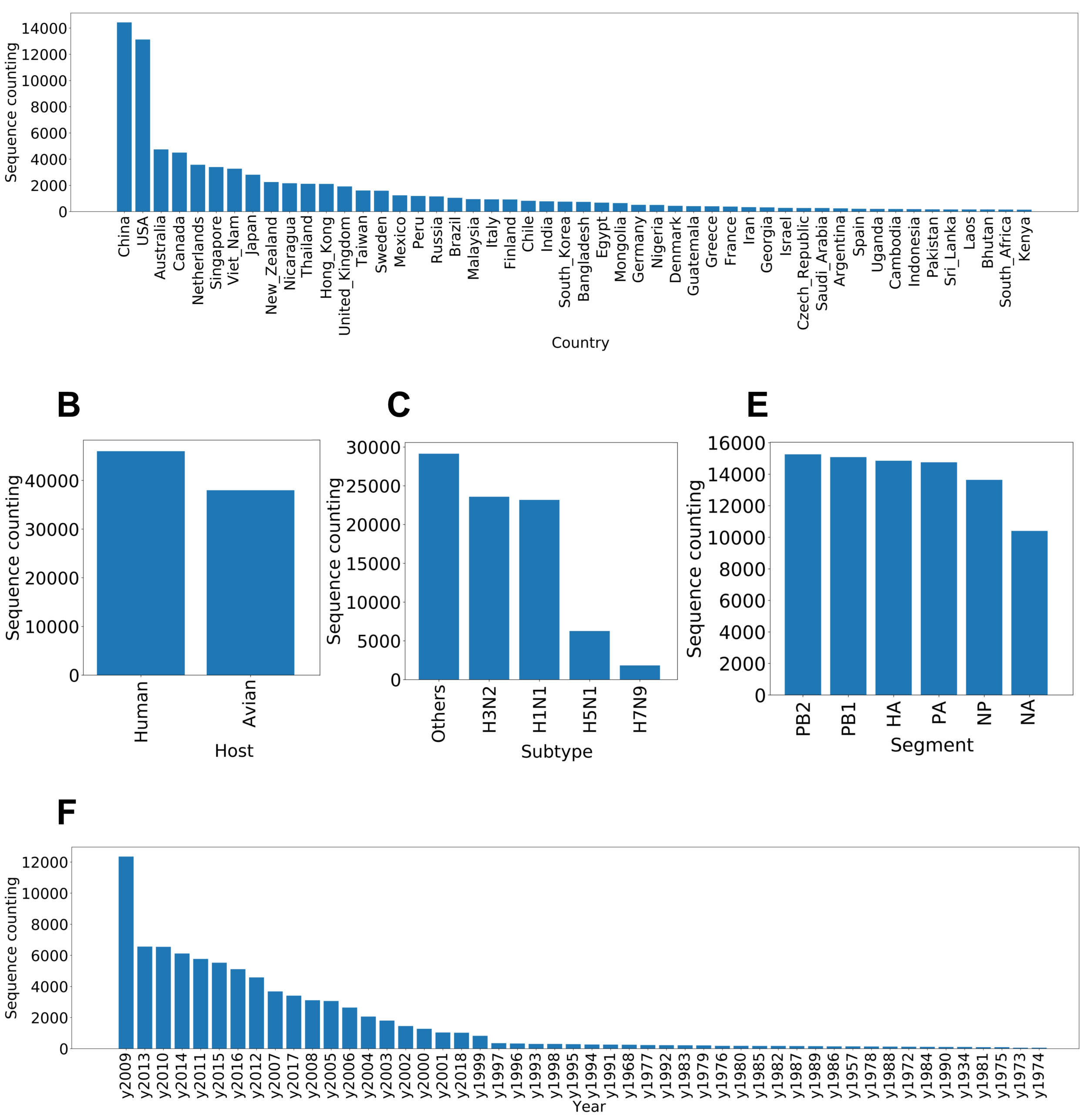

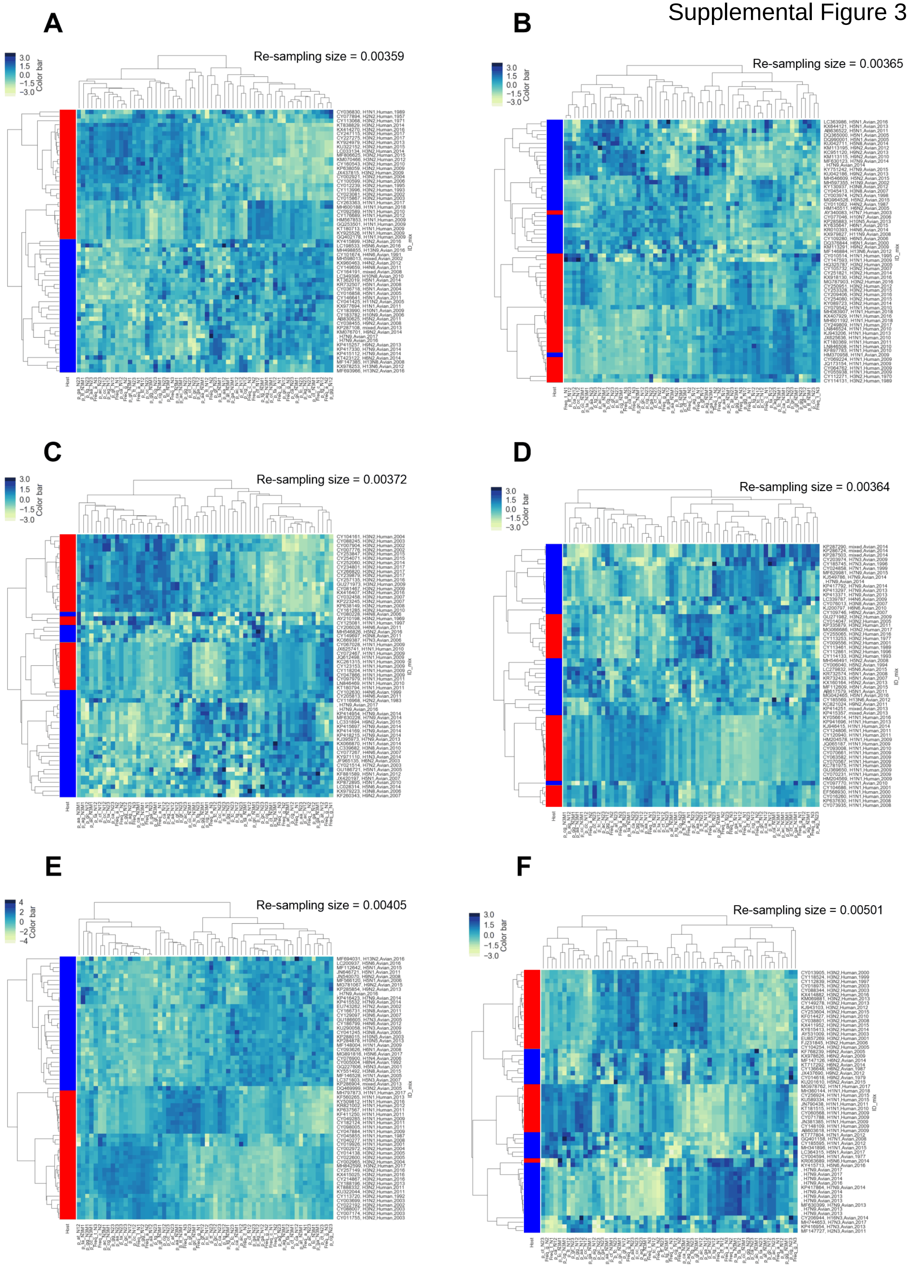

Supplemental Figure 3

Supplemental Figure 4

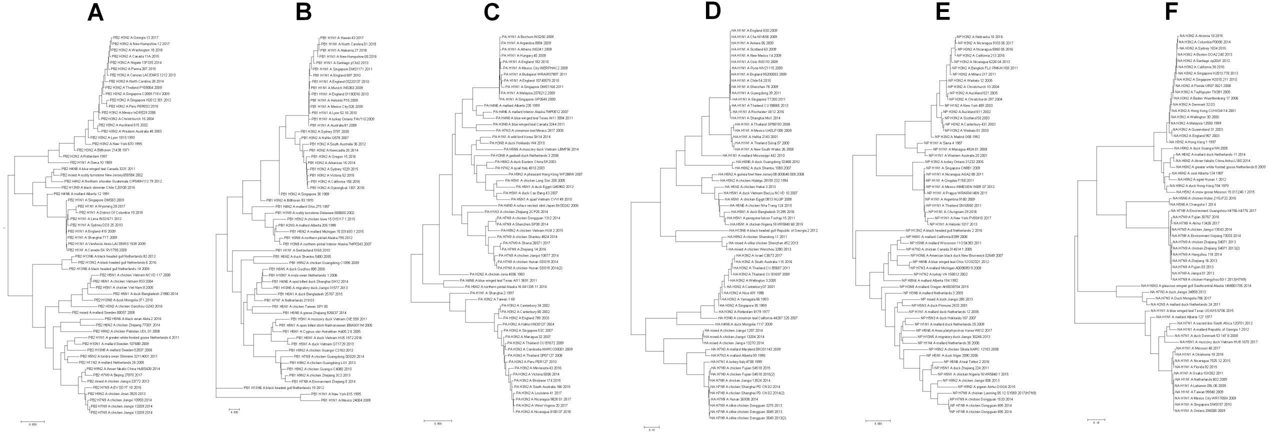

Supplemental Figure 5

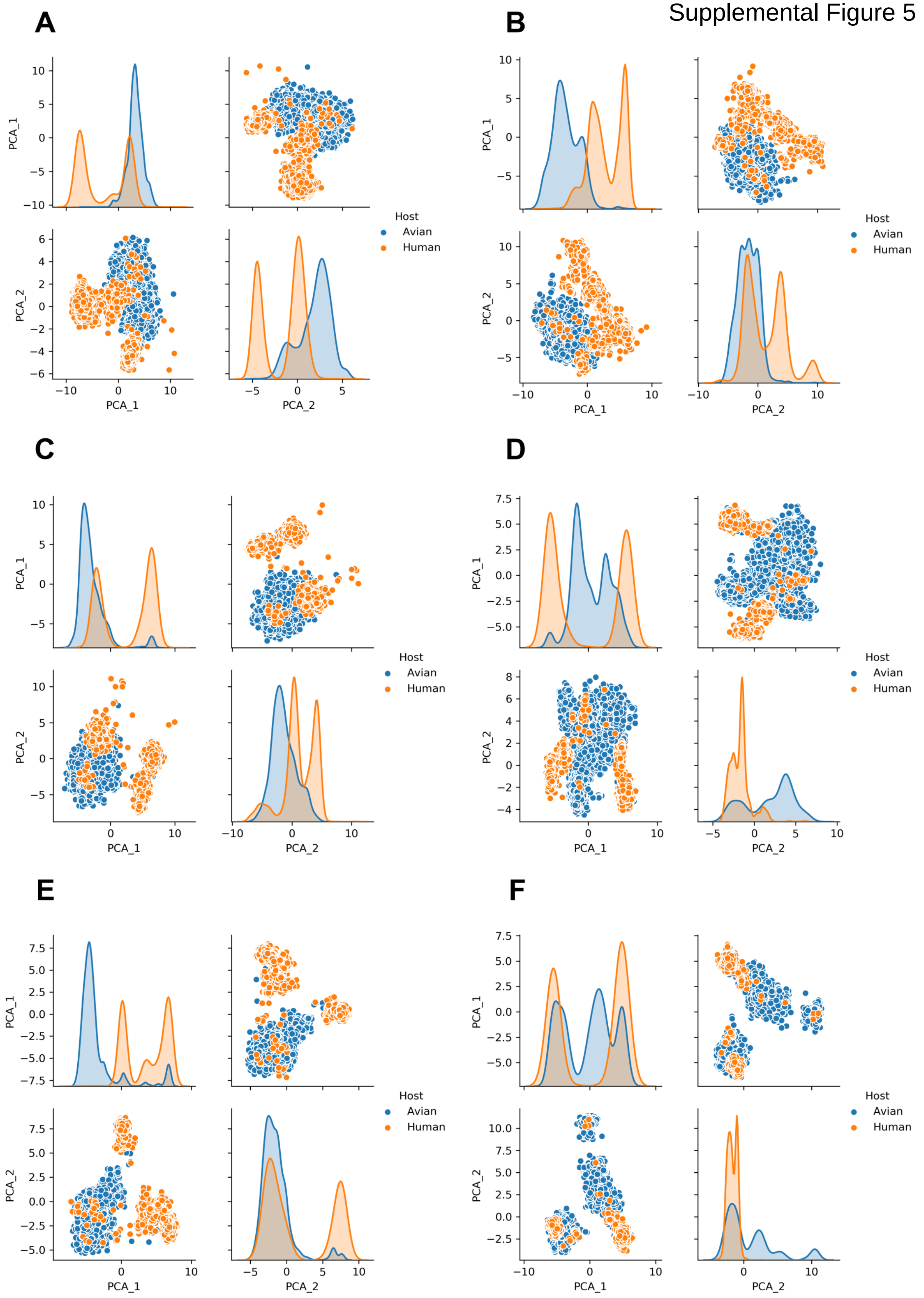

Supplemental Figure 6

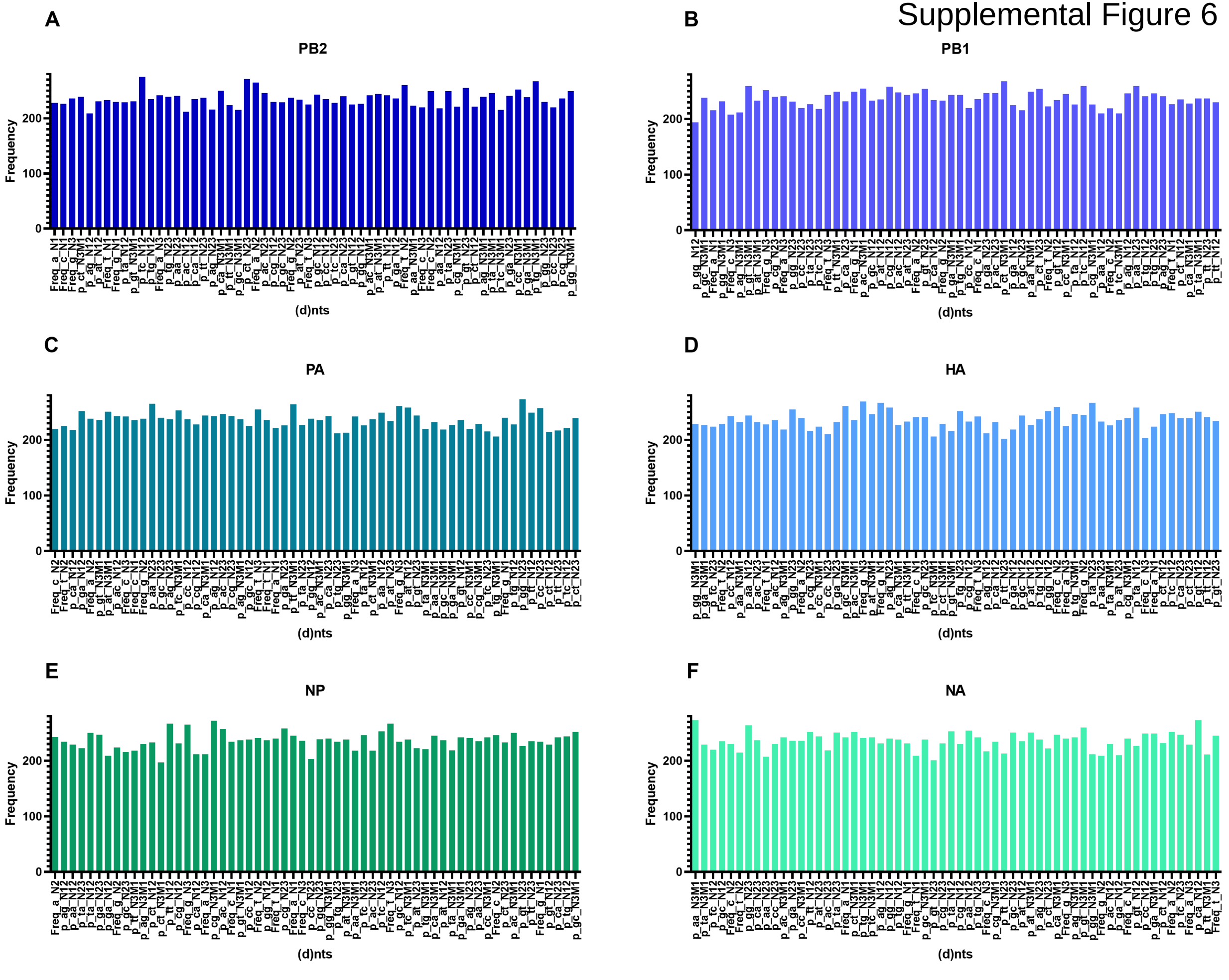

Supplemental Figure 7

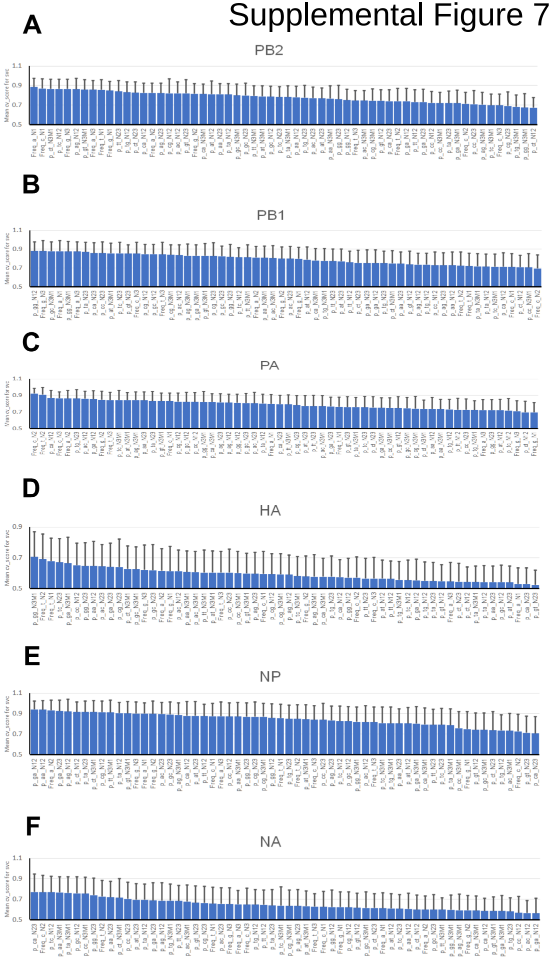

Supplemental Figure 8

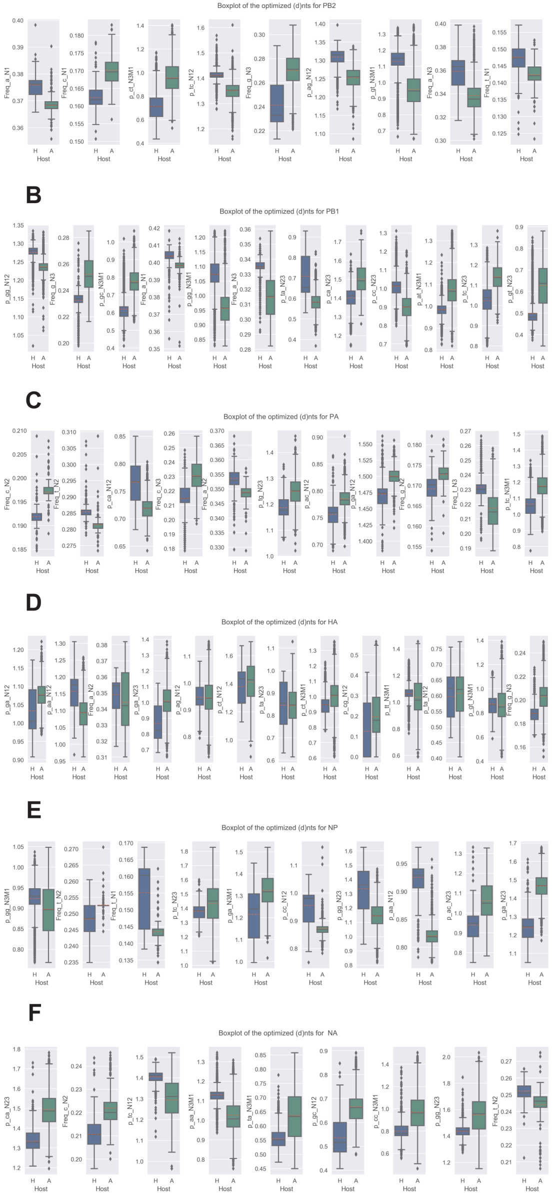

Supplemental Figure 9

A

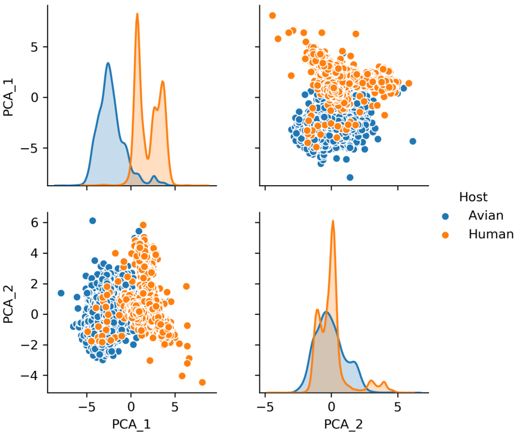

B

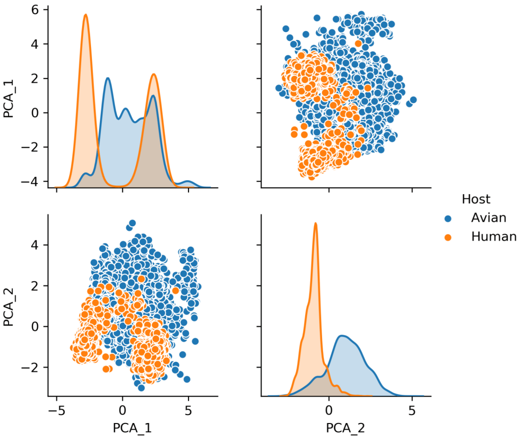

Supplemental Figure 10

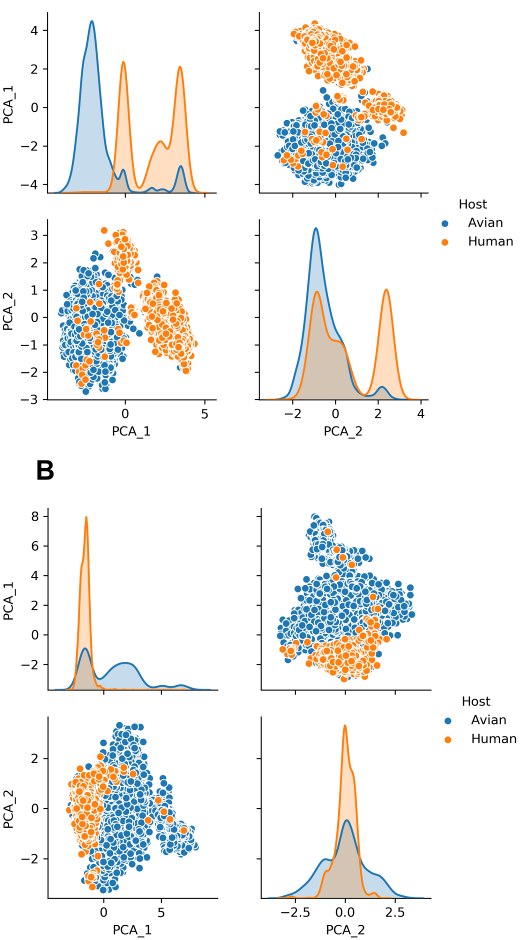

Supplemental Figure 10

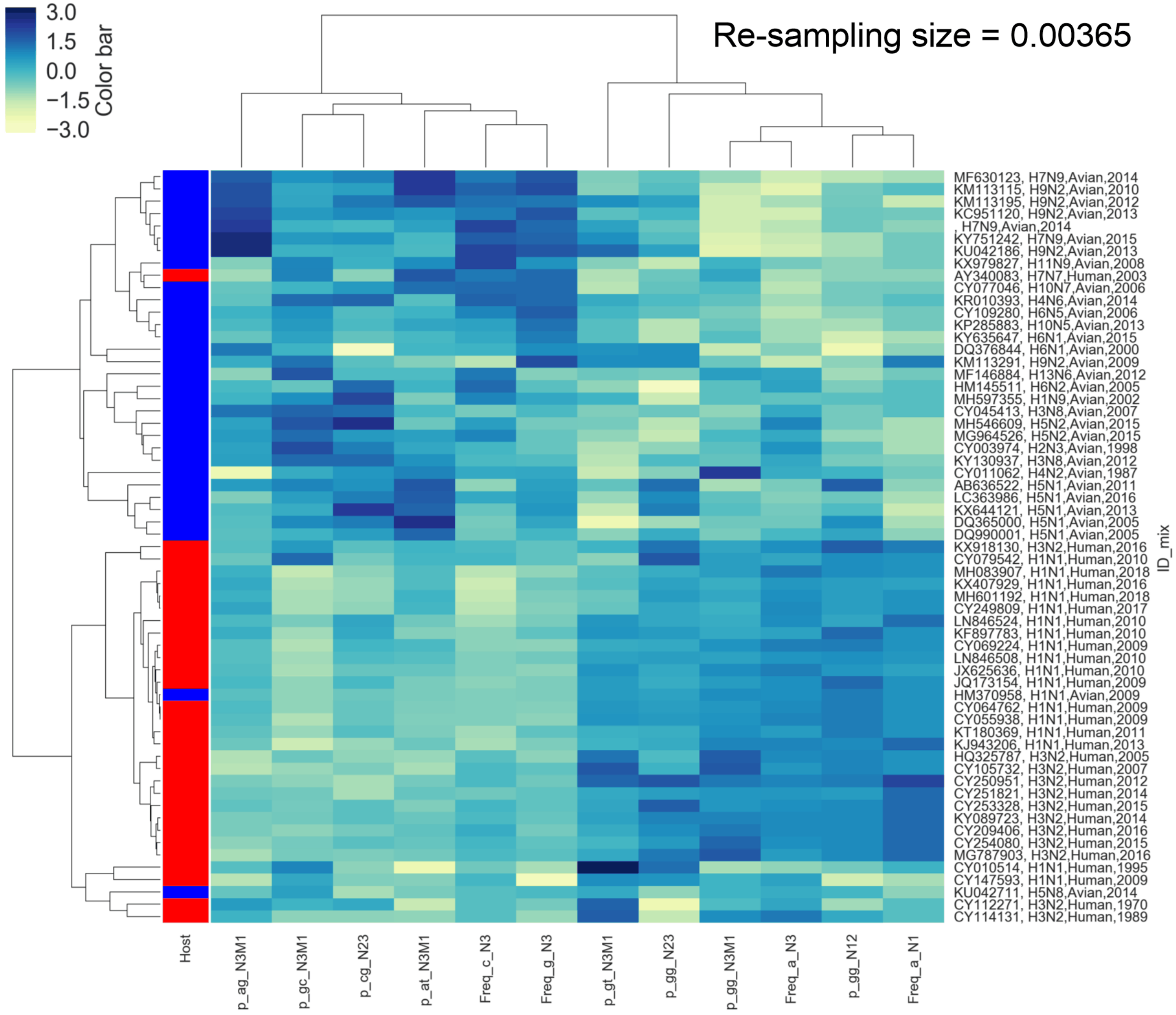

Supplemental Figure 11

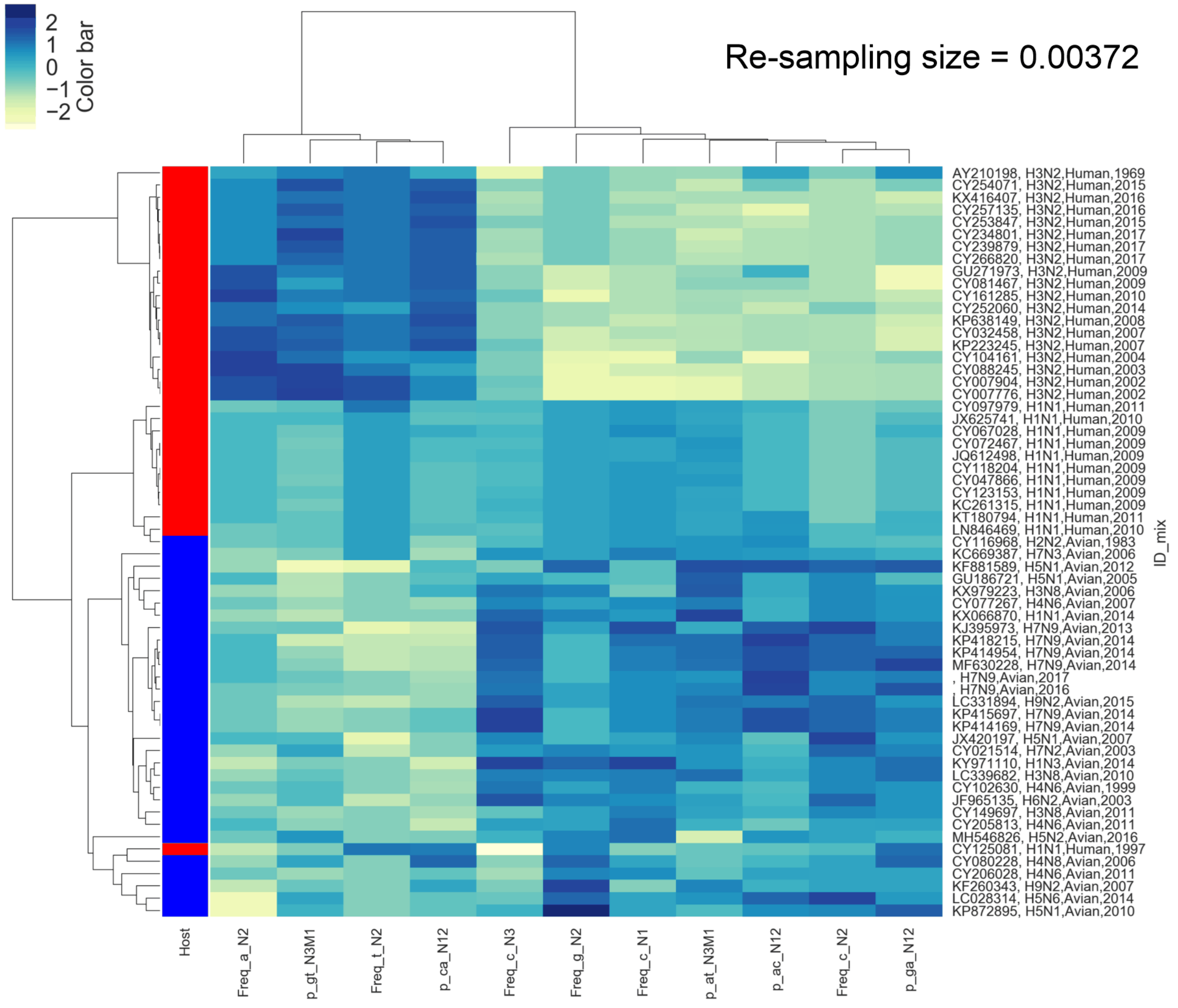

Supplemental Figure 13

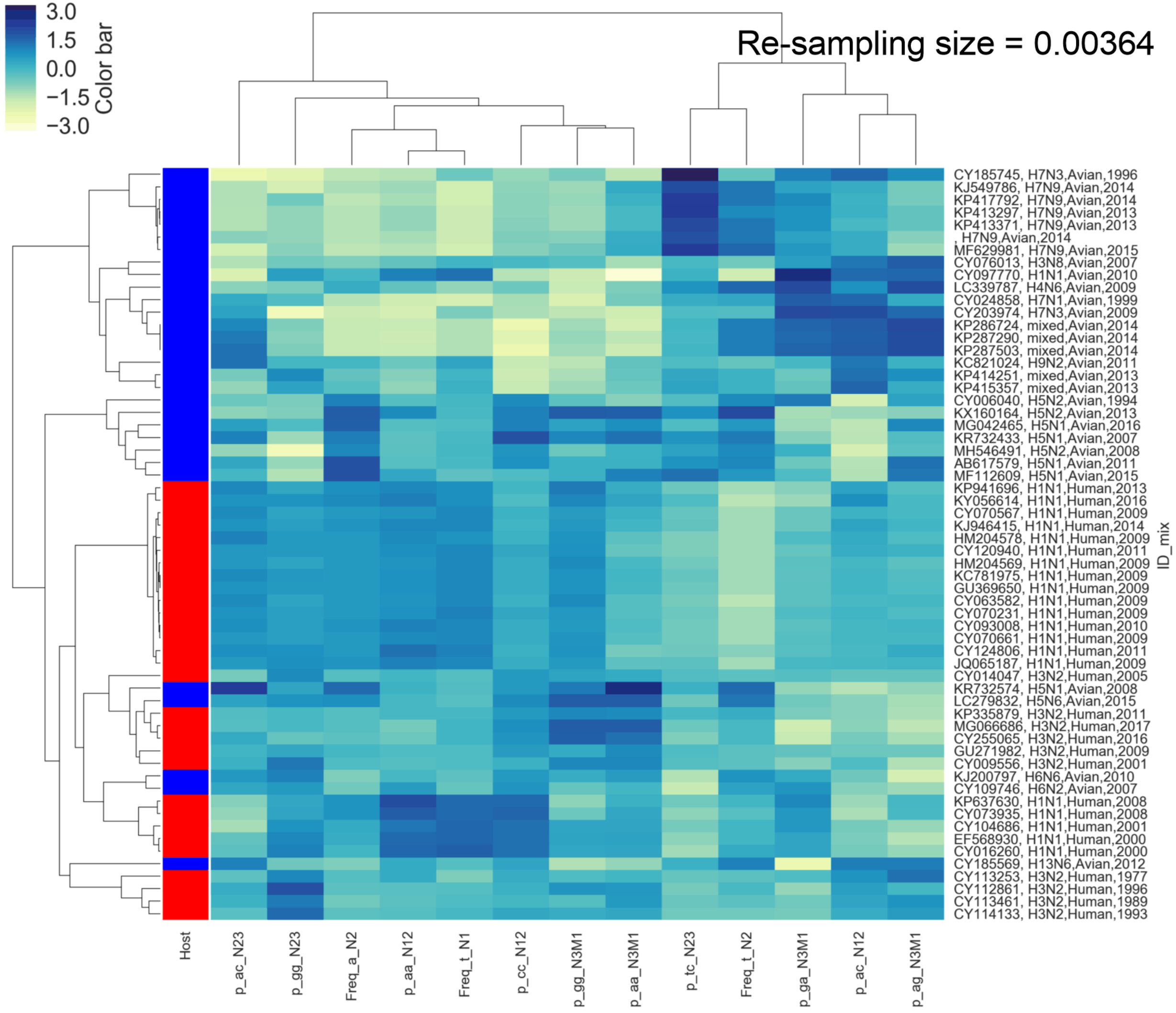

Supplemental Figure 14

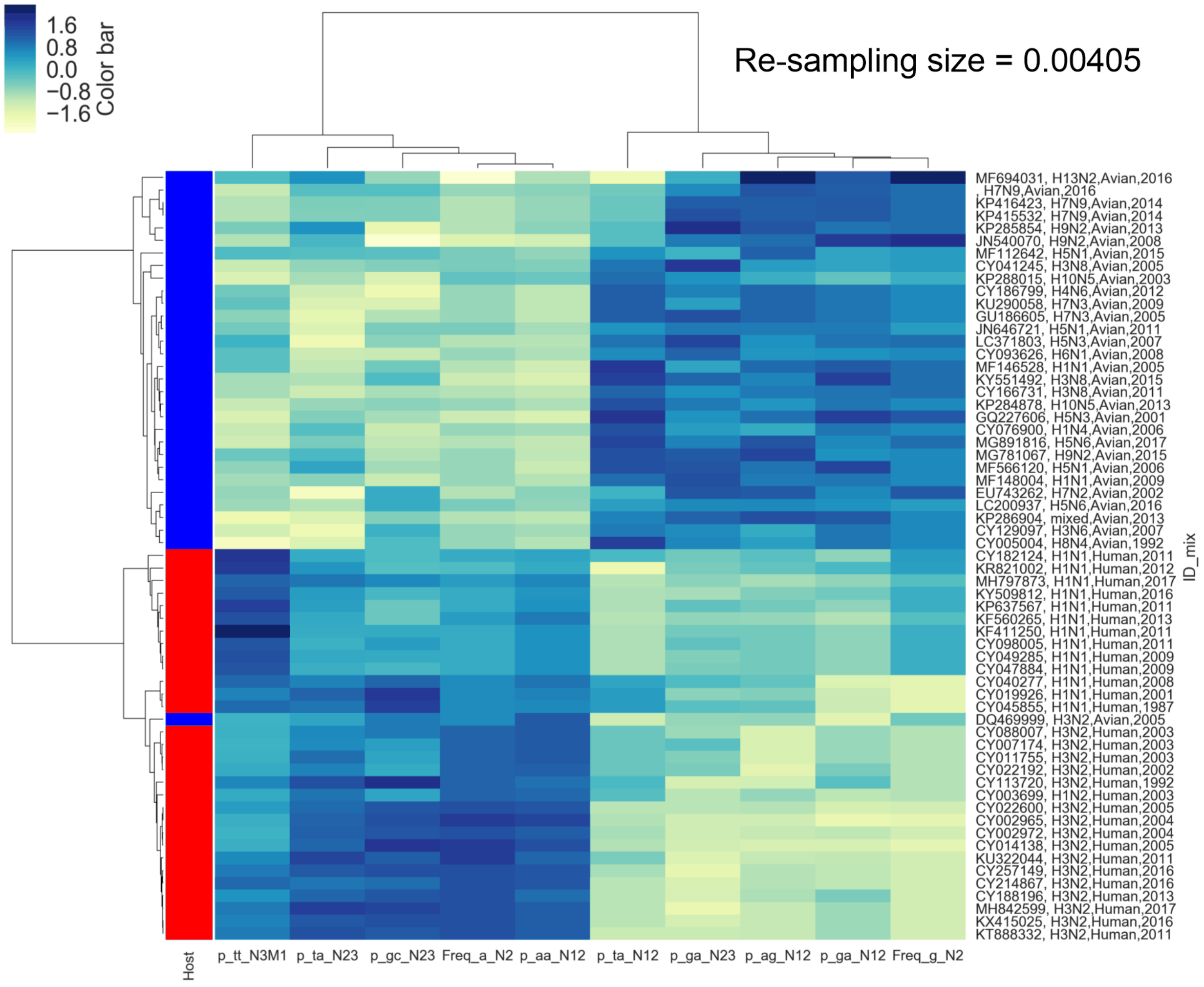

Supplemental Figure 15

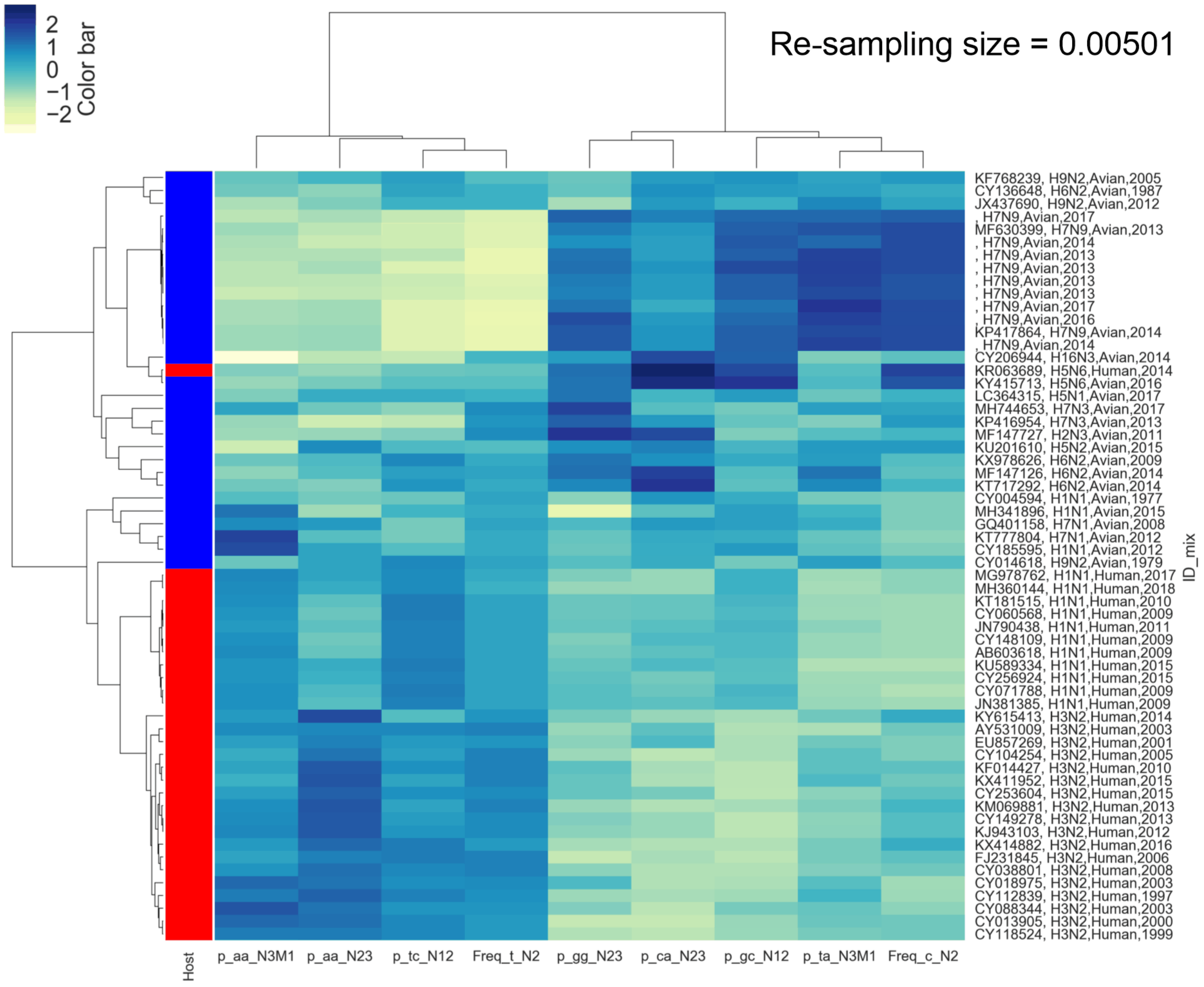

Supplemental Figure 16

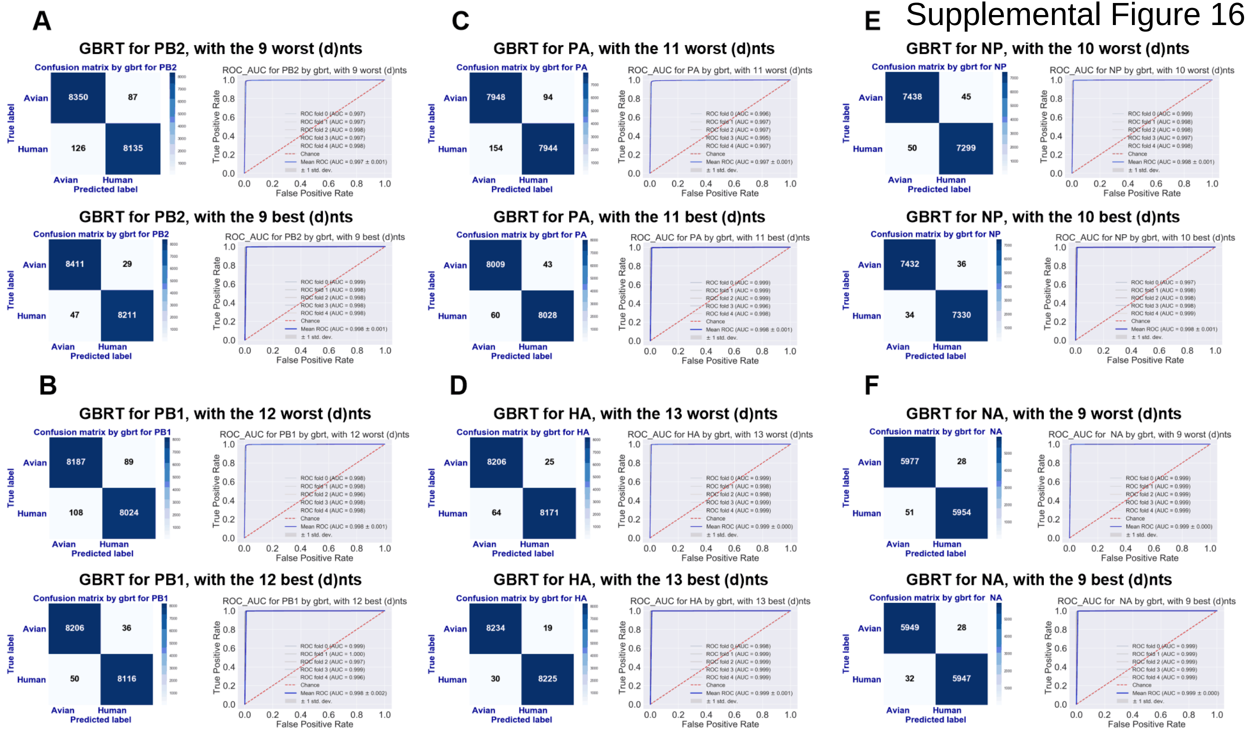

Supplemental Figure 17

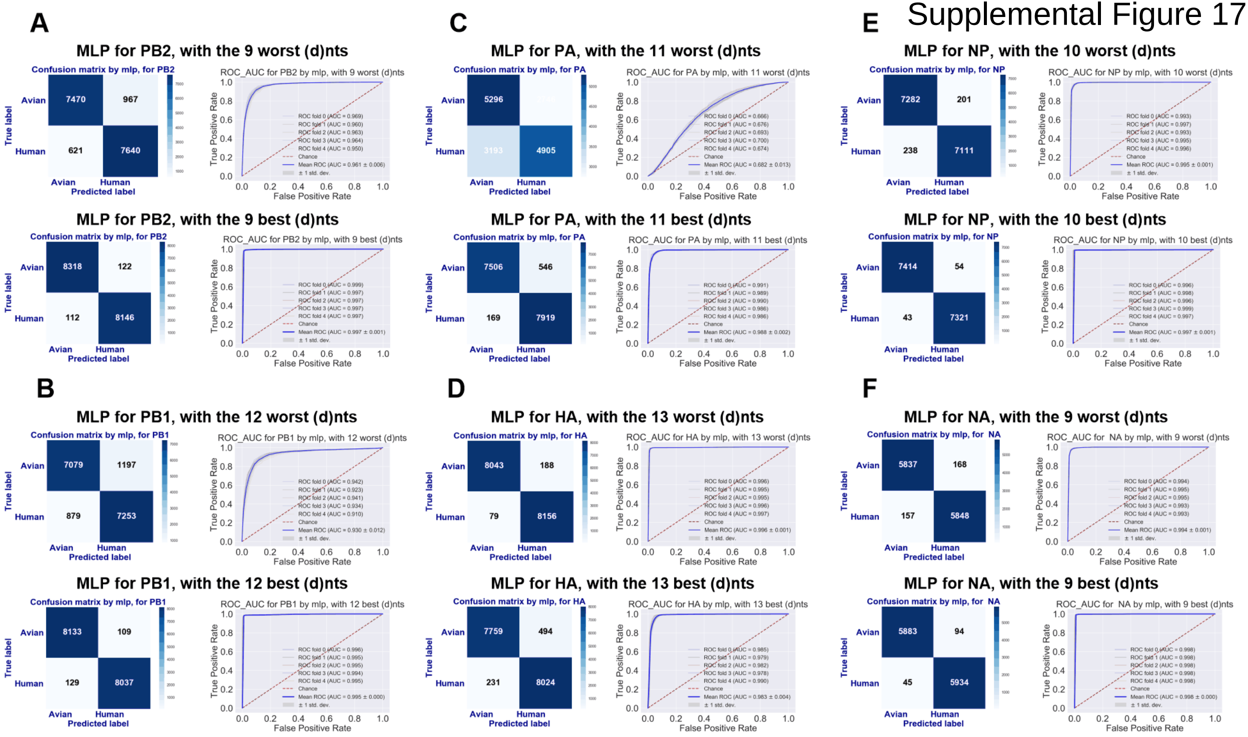

Supplemental Figure 18

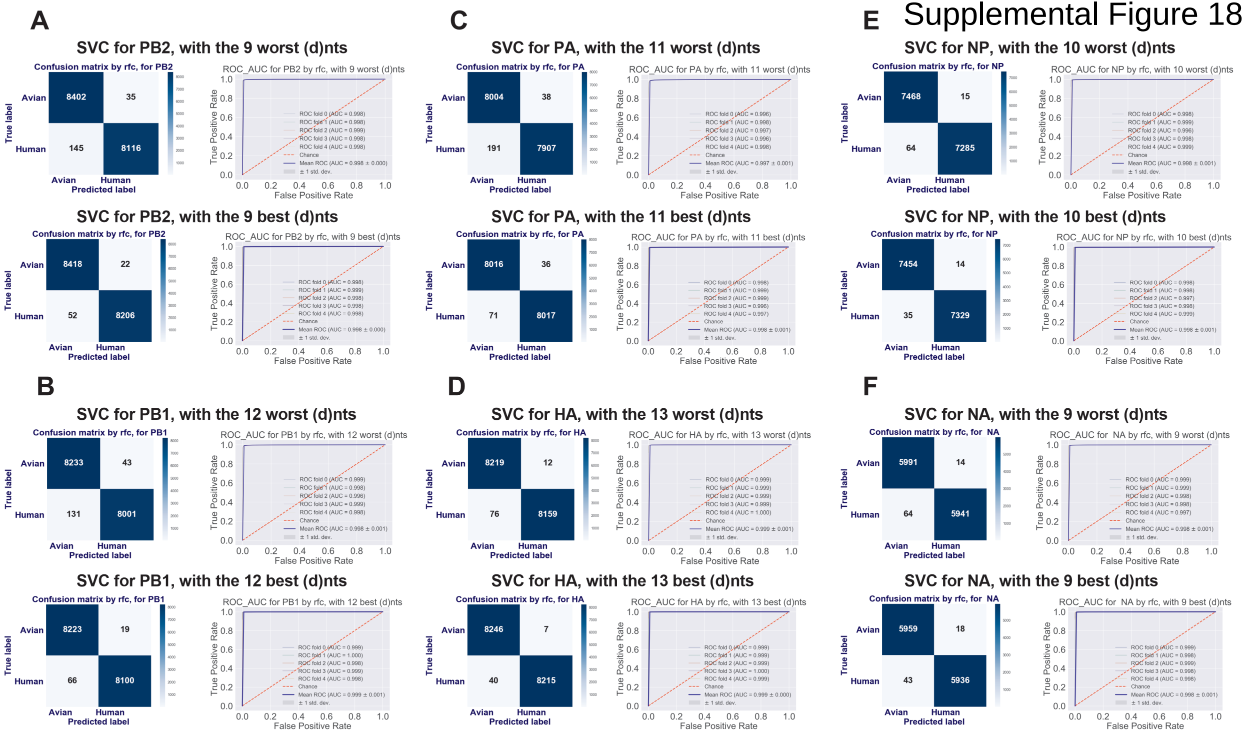

Supplemental Figure 19

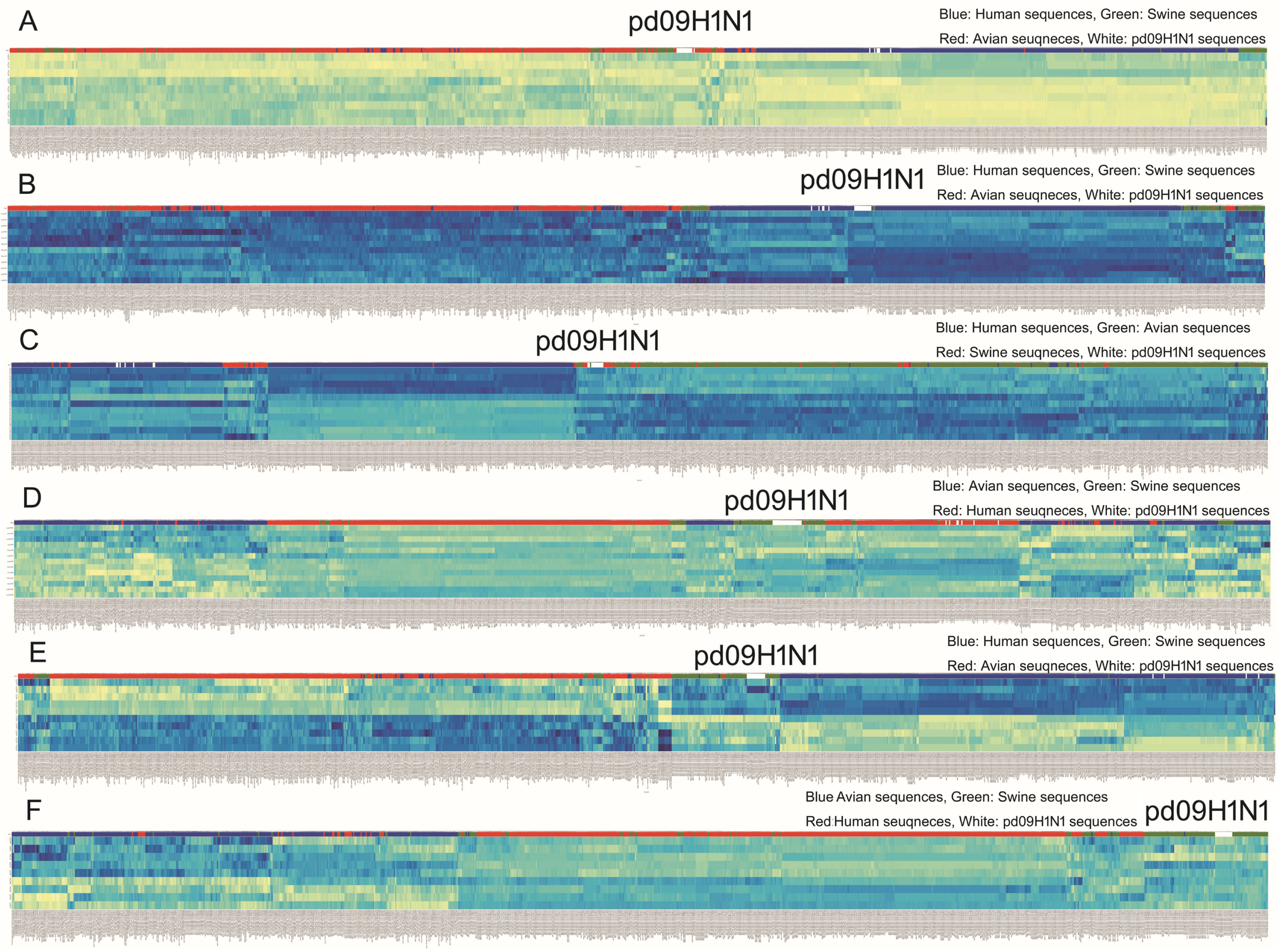

Supplement: msz276_Supplementary_Data [file msz276_supplementary_data.pdf]
